# Global burden trends and menopause timing associations for gynecological and breast cancers in postmenopausal women

**DOI:** 10.1016/j.isci.2026.115967

**Published:** 2026-06-01

**Authors:** Shuxin Li, Yu Tian, Cui Chen, Dengyi Duan, Jinhua Yan, Tao Zeng

**Affiliations:** 1 Department of Medical Oncology, Sichuan Cancer Hospital & Institute, Sichuan Cancer Center, School of Medicine, University of Electronic Science and Technology of China, Chengdu, China; 2 Department of Radiation Oncology, Precision Radiation in Oncology Key Laboratory of Sichuan Province, Sichuan Clinical Research Center for Cancer, Sichuan Cancer Hospital & Institute, Sichuan Cancer Center, University of Electronic Science and Technology of China, Chengdu, China; 3 Guangdong Cardiovascular Institute, Guangdong Provincial People’s Hospital, Guangdong Academy of Medical Sciences, Guangzhou, Guangdong Province, China; 4 Department of Radiology, Sichuan Clinical Research Center for Cancer, Sichuan Cancer Hospital & Institute, Sichuan Cancer Center, Affiliated Cancer Hospital of University of Electronic Science and Technology of China, Chengdu, China; 5 Department of General Internal Medicine, Sichuan Clinical Research Center for Cancer, Sichuan Cancer Hospital &Institute, Sichuan Cancer Center, University of Electronic Science and Technology of China, Chengdu, China

**Keywords:** Health sciences, Medicine, Medical specialty, Internal medicine, Oncology, Reproductive medicine

## Abstract

With women aged ≥50 years projected to reach 1.65 billion by 2050, gynecological and breast cancers impose an escalating postmenopausal burden. Whether menopausal timing independently influences cancer risk remains uncertain. We integrated Global Burden of Disease data (204 countries, 1990–2021) with Mendelian randomization and Bayesian projections to evaluate causality and forecast trajectories. Breast and uterine cancer incidence increased (0.17%–0.47% annually), while cervical and ovarian declined, with regional inequalities persisting. By 2040, absolute cases and deaths will surge 60%–129% despite stable age-standardized rates due to population aging. Mendelian randomization using 114 genetic variants from 143,819 women suggests that later menopause increases the risk of breast (odds ratio [OR], 1.24, 95% confidence interval [CI], 1.11–1.39), endometrial (OR, 1.49; 95% CI, 1.23–1.82), and ovarian cancers (OR, 1.23; 95% CI, 1.09–1.39), but not cervical cancer, confirming distinct human papillomavirus (HPV)-driven etiology. Population aging will nearly double cancer deaths by 2040, demanding healthcare expansion, HPV vaccination, and risk-stratified screening.

## Highlights

• Gynecological and breast cancer burden rising globally in postmenopausal women
• Cervical cancer concentrated in low-SDI regions with widening health inequalities
• Later menopause increases breast, endometrial, and ovarian cancer risk
• No causal association found between menopausal age and cervical cancer risk

## Graphical abstract

**Figure:**
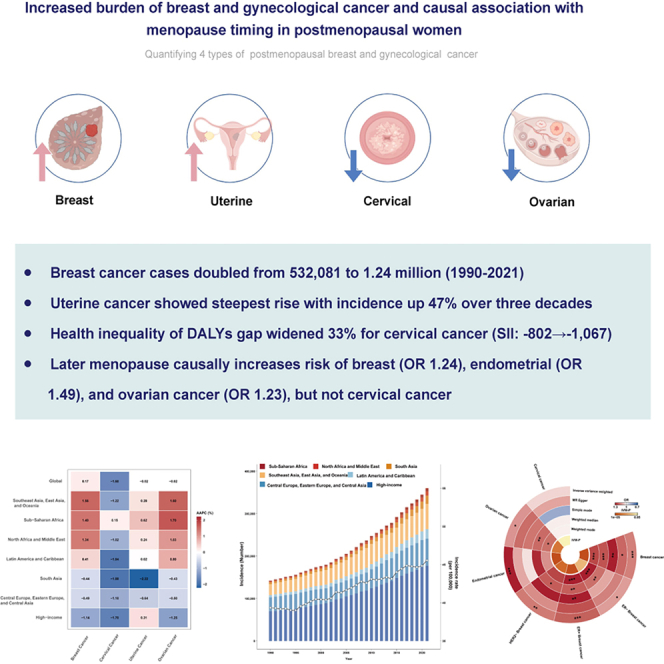

## Introduction

According to the United Nations, the global population of women aged 50 years and older is projected to increase from 985 million in 2020 to 1.65 billion by 2050, with women aged 50 and over accounting for 26% of all women and girls globally in 2021.^1^^,^^2^ This demographic shift underscores the urgent need to understand the long-term health consequences of menopause, a pivotal transition marked by the cessation of ovarian function.^3^ Menopause, typically occurring around age 45–55 years,^1^ marks a critical biological transition characterized by dramatic hormonal changes, after which women spend approximately one-third of their lives in the postmenopausal stage. This phase is associated with increased risks of multiple chronic diseases, with gynecological (including ovarian, cervical, and endometrial) and breast cancers representing a major and growing public health concern.^4^^,^^5^^,^^6^

Emerging evidence from observational epidemiology suggests that age at menopause—reflecting lifetime cumulative estrogen exposure—may play a pivotal role in gynecological and breast cancer etiology.^7^^,^^8^ For breast cancer, a landmark meta-analysis of 117 epidemiological studies encompassing 118,964 women with invasive breast cancer demonstrated that breast cancer risk increased by a factor of 1.029 (95% confidence interval [CI], 1.025–1.032) for every year older at menopause.^7^^,^^9^ For uterine cancer, a meta-analysis of 18 studies with 957,242 participants revealed a positive dose-response relationship, with a pooled relative risk of 1.89 (95% CI, 1.58–2.26) for highest versus lowest menopausal age, and cancer risk rising significantly when menopausal age exceeded 46.5 years.^10^ Associations with ovarian and cervical cancers are more complex. For cervical cancer, while the disease is primarily driven by human papillomavirus (HPV) infection, hormonal factors such as long-term oral contraceptive use have been associated with increased risk among HPV-infected women, although direct associations between age at menopause and cervical cancer risk remain inadequately characterized, with most evidence focusing on parity and contraceptive use rather than menopausal timing.^11^^,^^12^ Whether natural age at menopause independently affects ovarian cancer risk remains controversial. While some meta-analyses clearly link hormone replacement therapy (HRT) to increased risk, the evidence for natural menopause is far less conclusive.^5^^,^^13^ For instance, a large pooled analysis found only a weak, marginally significant association (hazard ratio = 1.09; 95% CI, 0.99–1.20; *p* = 0.06), suggesting any direct influence is likely minimal.^14^

However, evidence remains inconsistent. A recent National Health and Nutrition Examination Survey (NHANES) study (1999–2020, *n* = 8,219) paints a more complex picture, reporting a significant inverse correlation and a non-linear, L-shaped relationship where earlier menopausal age was unexpectedly associated with an increased risk for cervical, ovarian, and uterine cancers.^15^ Additionally, another retrospective study analysis consistently demonstrated inverse associations, with earlier menopause linked to higher cervical and ovarian cancer risk.^16^ These inconsistencies in observational epidemiology preclude definitive causal inference, as they are inherently limited by confounding from shared genetic, socioeconomic, lifestyle, and reproductive factors. Moreover, previous studies have generally focused on gynecological and breast cancers as a whole, without a comprehensive assessment of their disease burden.^17^ Specifically, data are lacking for women aged 55 years and older, who face unique risks due to postmenopausal hormonal changes, aging, and long-term carcinogenic exposures.

To address these multifaceted gaps, we conducted a two-stage integrated analysis. First, we comprehensively characterized the global burden (incidence, mortality, prevalence, and disability-adjusted life years [DALYs]) of breast, ovarian, cervical, and uterine cancers specifically focusing on women aged 55 and older to capture the fully postmenopausal population. The Global Burden of Disease (GBD) study, coordinated by the Institute for Health Metrics and Evaluation (IHME), represents the most comprehensive effort to systematically quantify health loss across diseases, injuries, and risk factors worldwide. GBD 2021 covers 371 diseases and injuries across 204 countries and territories from 1990 to 2021, drawing on standardized case definitions based on the International Classification of Diseases (ICD) coding system to ensure cross-country comparability. Disease burden is estimated through a rigorous modeling framework that integrates data from vital registration systems, cancer registries, household surveys, and published literature, with uncertainty intervals quantified at each stage to reflect data quality and heterogeneity. A key strength of the GBD framework is its capacity to generate comparable estimates across diverse health systems and data environments, enabling global and regional trend analyses that would otherwise be impossible. While prior GBD-based analyses have characterized the global burden of these cancers across all age groups,^17^^,^^18^ a key limitation is the systematic dilution of postmenopausal burden through age standardization, which obscures where disease burden actually concentrates. The present study addresses this gap by focusing specifically on women aged ≥55 years—a biologically distinct and rapidly growing demographic—and further extends existing evidence through Mendelian randomization (MR) analyses of menopausal timing and cancer risk.

Second, we employed MR analysis to investigate the causal effect of age at menopause on the risk of these four cancers, thereby elucidating the potential biological pathway underlying the observed epidemiological patterns. MR offers a powerful approach to overcome observational study limitations by leveraging genetic variants as instrumental variables.^19^^,^^20^ Because genetic variants are randomly allocated at conception and largely immune to reverse causation and confounding, MR provides more robust causal evidence than traditional observational approaches. Importantly, MR estimates in this context reflect the effects of genetically determined age at natural menopause and should be interpreted accordingly—they do not necessarily generalize to clinical scenarios involving intervention-induced changes in menopausal timing, such as surgical menopause or hormonal therapies. By integrating comprehensive epidemiological surveillance of the most vulnerable postmenopausal population with rigorous causal inference, this study provides a holistic understanding of gynecological and breast cancer burden in older women and identifies modifiable biological determinants to inform precision prevention strategies and equitable resource allocation in the era of global aging.

## Results

### Global trends in gynecological and breast cancers among postmenopausal women aged ≥55 years

**Figure 1:**
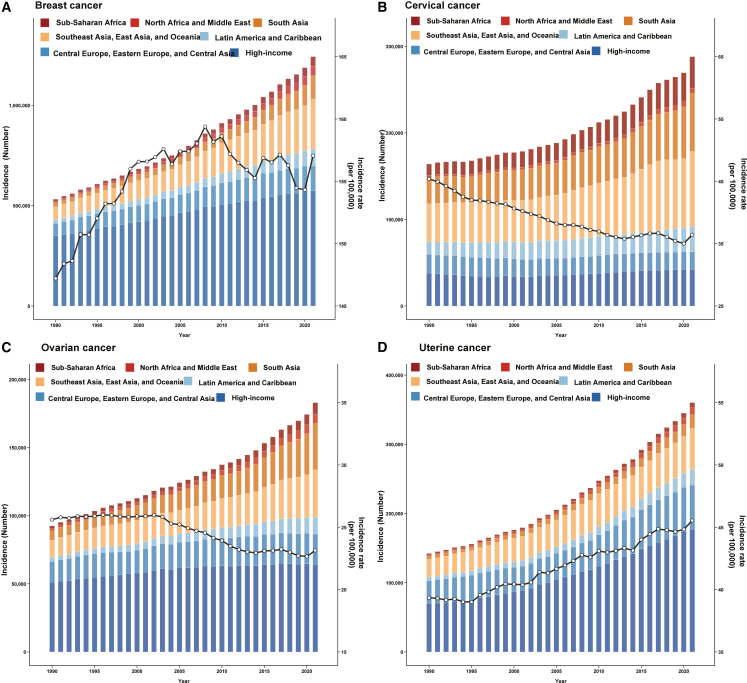
Global trends in absolute burden and incidence rates of gynecological and breast cancers among postmenopausal women aged ≥ 55 years (1990–2021) Stacked bars (left *y* axis) represent annual incident case counts by region, illustrating the growing absolute burden and its shifting geographic composition over time; the overlaid line (right *y* axis) depicts age-standardized incidence rates per 100,000, enabling direct comparison of absolute burden trends with underlying rate changes. (A) Breast cancer, (B) cervical cancer, (C) ovarian cancer, and (D) uterine cancer.

Globally, the burden of gynecological and breast cancers among postmenopausal women aged ≥55 years increased substantially from 1990 to 2021, with distinct patterns across cancer types (Tables S1 and S2). Breast cancer incident cases more than doubled (from 532,081 to 1,242,164), although incidence rates remained relatively stable (from 147.21 to 157.06 per 100,000; average annual percentage change [AAPC], 0.17%; 95% CI, 0.10–0.23; Figure 1A). Cervical cancer demonstrated global declining trends, with incidence rates decreasing from 45.38 to 36.43 per 100,000 (AAPC, −0.75%; Figure 1B). Ovarian cancer showed declining incidence rates despite rising absolute cases (from 92,579 to 183,007), with rates decreasing from 25.61 to 23.14 per 100,000 (AAPC, −0.38%; Figure 1C). Uterine cancer exhibited the most pronounced increasing trend, with incident cases more than doubling (from 142,221 to 360,187) and incidence rates rising from 39.35 to 45.54 per 100,000 (AAPC, 0.47%; Figure 1D). Prevalence patterns consistently mirrored incidence trends across all cancer types: breast cancer prevalent cases increased from 5.9 to 13.0 million with minimal rate change (AAPC, 0.01%), while other cancers showed similar concordance between incidence and prevalence trajectories (Figure S1). Detailed annual percentage change (APC) trends by year for each cancer type and region are presented in Table S3.

**Figure 2:**
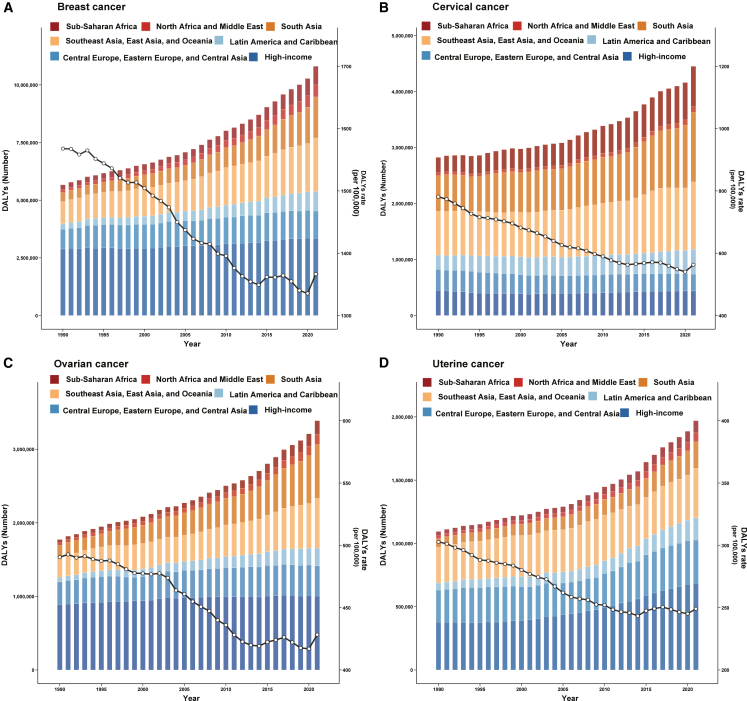
Global trends in absolute DALYs and DALY rates of gynecological and breast cancers among postmenopausal women aged ≥ 55 years (1990–2021) Stacked bars (left *y* axis) represent annual DALY counts by region, reflecting the growing absolute disease burden and its shifting geographic composition; the overlaid line (right y axis) depicts age-standardized DALY rates per 100,000. (A) Breast cancer, (B) cervical cancer, (C) ovarian cancer, and (D) uterine cancer.

Global DALY and mortality burdens increased substantially in absolute numbers for all cancers, although rates showed divergent patterns (Tables S4 and S5). Breast cancer DALY rates declined from 1,567.83 to 1,366.48 per 100,000 (AAPC, −0.49%; Figure 2A), driven by reduced mortality (from 66.09 to 59.04 per 100,000; AAPC, −0.13%). Cervical cancer showed the most pronounced DALY decline, with rates decreasing from 781.33 to 562.75 per 100,000 (AAPC, −1.10%; Figure 2B). Ovarian cancer DALY rates declined modestly from 302.76 to 249.05 per 100,000 (AAPC, −0.50%; Figure 2C). Uterine cancer DALY rates remained relatively stable, declining marginally from 429.47 to 428.32 per 100,000 (AAPC, −0.64%; Figure 2D). Death patterns consistently mirrored DALY trends across all cancer types (Figure S2).

### Regional variations in disease burden

**Figure 3:**
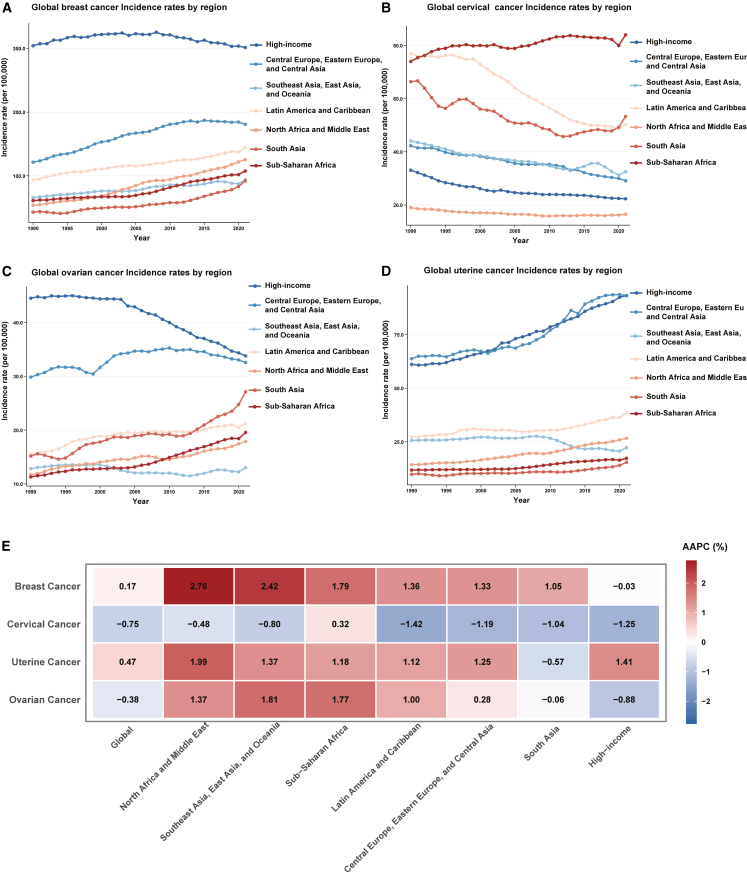
Regional trends in age-standardized incidence rates of gynecological and breast cancers among postmenopausal women aged ≥ 55 years (1990–2021) (A–D) Region-specific incidence rates (per 100,000) for (A) breast cancer, (B) cervical cancer, (C) ovarian cancer, and (D) uterine cancer across seven global regions, complementing the global aggregate perspective presented in Figure 1. (E) Heatmap displaying the average annual percentage change (AAPC) in incidence rates across cancer types and regions.

Regional disparities in incidence rates and trends were pronounced across all cancer types (Figure 3; Tables S1 and S2). For breast cancer, high-income regions had the highest rates (301.36 per 100,000) with slight declines (AAPC, −0.03%), while North Africa/Middle East (AAPC, 2.76%) and Southeast Asia/East Asia/Oceania (AAPC 2.42%) showed steep increases. Sub-Saharan Africa demonstrated the most rapid acceleration (AAPC, 1.79%), rising from 61.01 to 107.62 per 100,000 (Figure 3A). For cervical cancer, sub-Saharan Africa bore the highest burden and was the only region with increasing rates (from 73.97 to 84.01 per 100,000; AAPC, 0.32%), while high-income regions showed the steepest declines (AAPC, −1.19%; Figure 3B). Ovarian cancer showed that high-income regions had the highest rates but significant declines (AAPC, −0.88%), while Southeast Asia/East Asia/Oceania (AAPC, 1.81%) and Sub-Saharan Africa (AAPC, 1.77%) exhibited substantial increases (Figure 3C). For uterine cancer, Central Europe/Eastern Europe/Central Asia (93.23 per 100,000; AAPC 1.25%) and high-income regions (93.13 per 100,000; AAPC 1.41%) had the highest rates, while North Africa/Middle East showed the steepest increase (AAPC, 1.99%; Figures 3D and 3E). Regional prevalence patterns consistently mirrored incidence trends (Figure S3).

**Figure 4:**
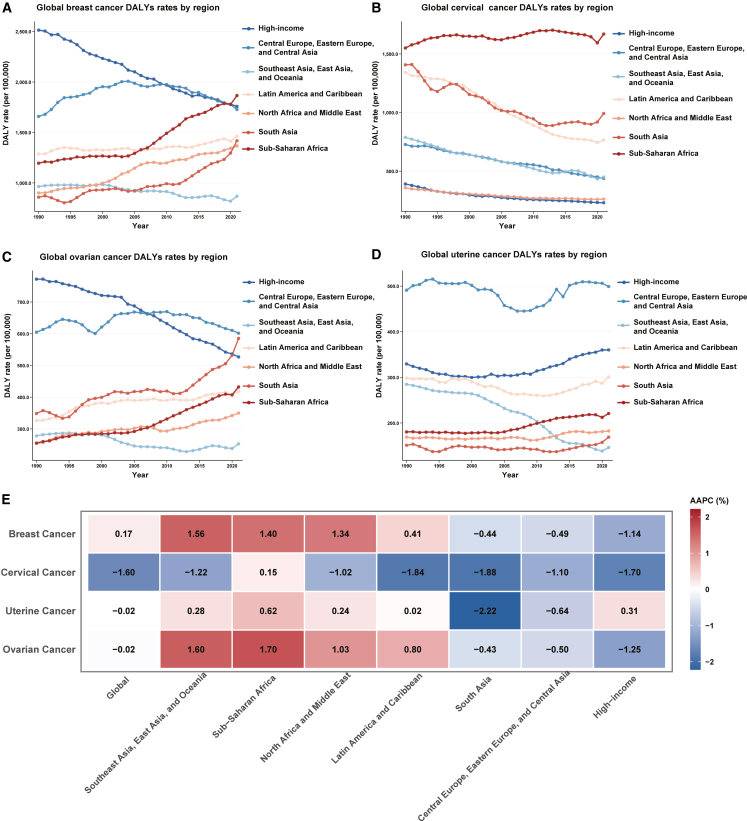
Regional trends in age-standardized DALY rates of gynecological and breast cancers among postmenopausal women aged ≥ 55 years (1990–2021) (A–D) Region-specific DALY rates (per 100,000) for (A) breast cancer, (B) cervical cancer, (C) ovarian cancer, and (D) uterine cancer across seven global regions, complementing the global aggregate perspective presented in Figure 2. (E) Heatmap displaying the AAPC in DALY rates across cancer types and regions.

Regional inequalities in DALYs and mortality were substantial (Figure 4; Tables S4 and S5). For breast cancer, high-income regions demonstrated the steepest DALY decline (AAPC, −1.14%), while Sub-Saharan Africa (AAPC, 1.40%), Southeast Asia/East Asia/Oceania (AAPC, 1.56%), and North Africa/Middle East (AAPC, 1.34%) showed substantial increases (Figure 4A). Cervical cancer showed that sub-Saharan Africa was the only region with increasing DALY rates (AAPC, 0.15%), maintaining rates exceeding 1,669 per 100,000—10-fold higher than high-income regions (230.63 per 100,000; Figure 4B). For ovarian cancer, high-income regions showed significant DALY declines (AAPC, −1.25%), while sub-Saharan Africa (AAPC, 1.70%) and Southeast Asia/East Asia/Oceania (AAPC, 1.60%) exhibited substantial increases (Figure 4C). Uterine cancer showed that Central Europe/Eastern Europe/Central Asia had the highest DALY rates (601.59 per 100,000; AAPC, −0.02%), while South Asia showed the steepest decline (AAPC, −2.12%; Figures 4D and 4E). Regional mortality patterns consistently mirrored DALY trends (Figure S4).

### Association between sociodemographic index and cancer burden

**Figure 5:**
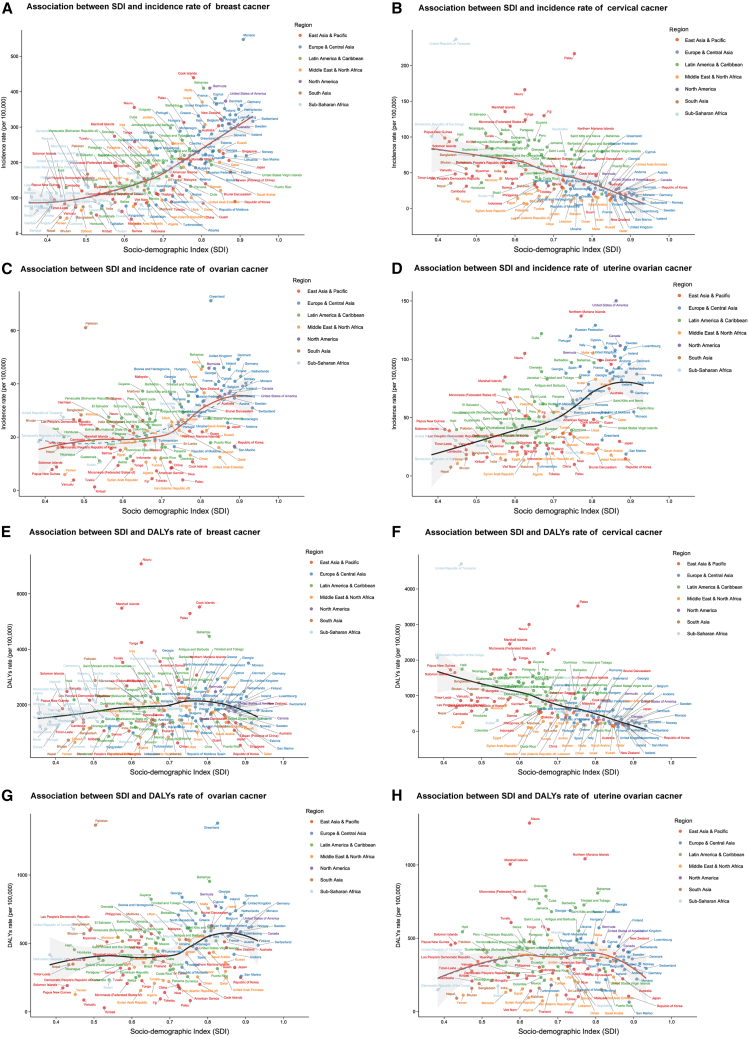
Association between SDI and cancer burden among postmenopausal women aged ≥ 55 years across 204 countries and territories (A–D) Associations between SDI and incidence rates for (A) breast cancer, (B) cervical cancer, (C) ovarian cancer, and (D) uterine cancer. (E–H) Associations between SDI and DALY rates for (E) breast cancer, (F) cervical cancer, (G) ovarian cancer, and (H) uterine cancer.

The relationship between sociodemographic development and cancer burden varied substantially across cancer types and health indicators (Figure 5; Table S6). Incidence rates demonstrated diverse associations with sociodemographic index (SDI) across cancer types (Figures 5A–5D). Breast cancer showed a strong quadratic association (R^2^ = 0.559), peaking at >300 per 100,000 in high-income countries. Cervical cancer demonstrated an inverse linear relationship (R^2^ = 0.206), with rates declining as development increased; sub-Saharan Africa bore rates >3,000 per 100,000 versus <500 in high-SDI regions. Ovarian cancer exhibited a positive linear correlation (R^2^ = 0.268), reflecting increased detection in higher-SDI countries. Uterine cancer showed a positive linear association (R^2^ = 0.336), with rates increasing substantially with development. Prevalence patterns largely mirrored incidence trends (Figure S5): breast cancer demonstrated the strongest quadratic association (R^2^ = 0.702), cervical cancer showed a weaker quadratic pattern (R^2^ = 0.180), and both ovarian cancer (R^2^ = 0.361) and uterine cancer (R^2^ = 0.408) exhibited positive linear relationships with increasing strength. DALY rates showed markedly different associations with SDI compared to incidence patterns (Figures 4E–4H). Breast cancer exhibited a weak linear correlation (R^2^ = 0.052), indicating improved outcomes despite rising incidence in high-SDI countries. Cervical cancer demonstrated the strongest inverse linear association (R^2^ = 0.277), with DALY rates declining substantially with increasing development. Ovarian cancer showed a positive linear correlation (R^2^ = 0.117), reflecting treatment access barriers in resource-limited settings alongside improved detection in higher-SDI countries. Uterine cancer displayed minimal correlation (R^2^ = 0.011), suggesting relatively stable outcomes across development levels. Mortality patterns consistently mirrored DALY trends (Figure S6): breast cancer showed a weak positive correlation (R^2^ = 0.123), cervical cancer demonstrated a strong inverse relationship (R^2^ = 0.252), ovarian cancer exhibited a positive correlation (R^2^ = 0.235), and uterine cancer showed minimal association (R^2^ = 0.042).

### Health inequalities in cancer burden across the sociodemographic spectrum

**Figure 6:**
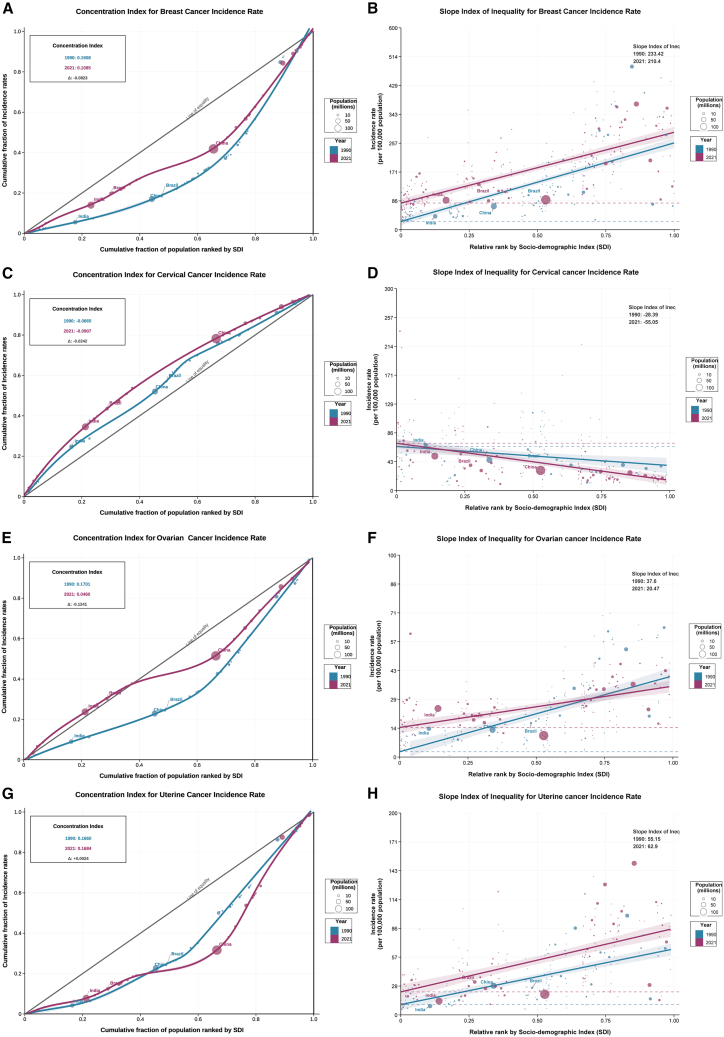
Health inequalities in gynecological and breast cancer incidence rates among postmenopausal women aged ≥ 55 years by sociodemographic index (A and B) (A) Concentration curves showing the cumulative distribution of breast cancer incidence by population ranked by sociodemographic index (SDI), and (B) slope indices of inequality showing the absolute rate difference in breast cancer incidence (per 100,000 women aged ≥55 years) across the SDI gradient (ranging from 0 to 1). (C and D) (C) Concentration curves and (D) slope indices of inequality for cervical cancer. (E and F) (E) Concentration curves and (F) slope indices of inequality for ovarian cancer. (G and H) (G) Concentration curves and (H) slope indices of inequality for uterine cancer.

Health inequalities in gynecological and breast cancers, measured by concentration and slope indices, revealed divergent patterns across cancer types (Table S7). Figure 6A presents the concentration curves for breast cancer incidence in 1990 and 2021. The concentration curve plots the cumulative share of the female population (aged ≥55 years), ranked from lowest to highest SDI along the *x* axis, against the cumulative share of breast cancer incidence burden on the *y* axis. A curve bowing below the diagonal line of equality indicates that breast cancer incidence is disproportionately concentrated among higher-SDI populations. In 1990, the concentration index was 0.19, reflecting substantial pro-rich concentration; by 2021, this had declined to 0.11, indicating a modest narrowing of relative inequality as breast cancer incidence increased in lower-SDI settings. Figure 6B presents the corresponding slope indices of inequality, illustrating that the absolute rate difference between the highest- and lowest-SDI extremes narrowed from 233.4 to 210.4 per 100,000 over the same period, reflecting convergence in absolute burden across the socioeconomic spectrum.

Prevalence inequalities similarly decreased (concentration index, 0.21–0.14; Figure S7), while mortality concentration shifted substantially (concentration index, 0.13–0.03; Figure S9). Most notably, DALY inequalities declined markedly (concentration index, 0.11–0.01; slope index of inequality [SII], 2,118.9–537.1 per 100,000; Figure S8), reflecting improved survival outcomes in lower-SDI settings. Cervical cancer burden remained disproportionately concentrated in lower-SDI populations, with widening absolute inequalities. Incidence slope indices increased from −28.4 to −55.1 per 100,000 (Figures 6C and 6D), while DALY inequalities intensified from −802.1 to −1,066.8 per 100,000 (Figure S8), indicating persistently greater disease burden among disadvantaged populations. Mortality inequalities similarly widened (SII, −26.6 to −40.6; Figure S9). Ovarian cancer showed declining inequalities across all metrics. Incidence concentration indices decreased from 0.17 to 0.05 (Figure 6E), with slope indices falling from 37.6 to 20.5 per 100,000 (Figure 6F). DALY inequalities similarly declined (concentration index, 0.14–0.01; SII, 670.5–239.1; Figure S8), suggesting more equitable distribution of disease burden. Uterine cancer incidence remained concentrated in higher-SDI populations, with concentration indices stable at 0.17 in both years (Figure 6G) and minimal change in absolute inequalities (SII, 55.2–62.9; Figure 6H). However, DALY inequalities narrowed substantially (SII, 204.8–71.3; Figure S8), indicating improved outcomes despite persistent incidence disparities.

### Projected global burden of gynecological cancers to 2040

**Figure 7:**
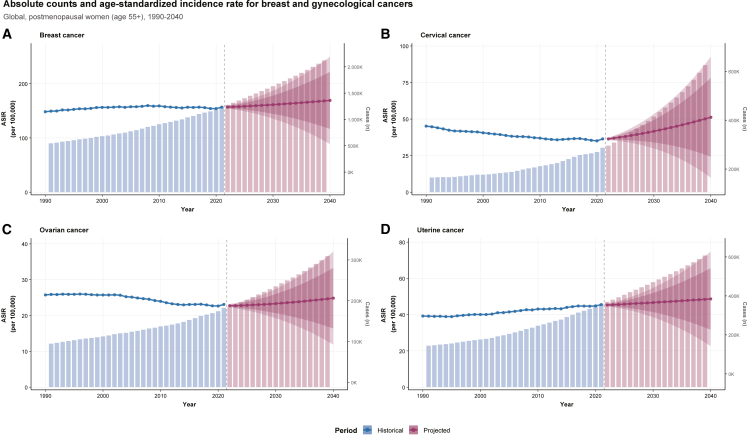
Projected global trends in age-standardized incidence rates of breast and gynecological and cancer among postmenopausal women to 2040 Age-standardized incidence rates (per 100,000) with projections for (A) breast cancer, (B) cervical cancer, (C) ovarian cancer, and (D) uterine cancer among women aged 55 years and older globally. Shaded areas indicate uncertainty intervals (UI): the innermost darkest shading represents 80% UI and the outermost lightest shading represents 95% UI.

**Figure 8:**
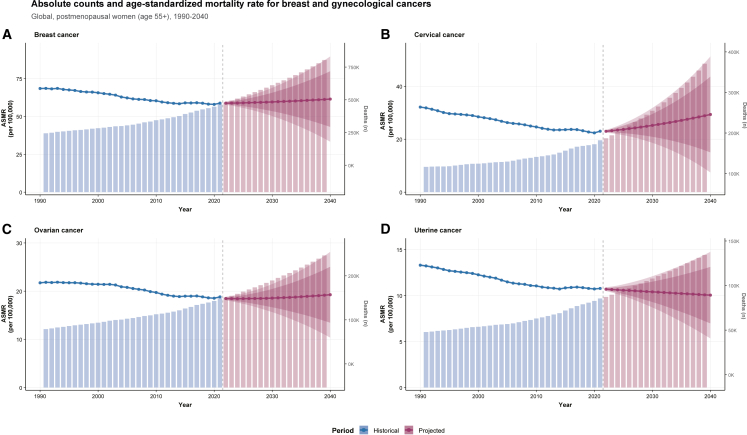
Projected global trends in age-standardized mortality rates and death counts of four gynecological cancers among postmenopausal women to 2040 Age-standardized mortality rates (per 100,000) and absolute death counts with projections for (A) breast cancer, (B) cervical cancer, (C) ovarian cancer, and (D) uterine cancer among women aged 55 years and older globally. Shaded areas indicate uncertainty intervals (UI): the innermost darkest shading represents 80% UI and the outermost lightest shading represents 95% UI.

Global age-standardized incidence rates among postmenopausal women are projected to increase for all four cancers by 2040 (Figure 7; Table S8). Cervical cancer shows the steepest rise (1.81% annually, from 36.4 to 51.2 per 100,000), followed by breast cancer (0.40% annually, 156.8–169.2), ovarian cancer (0.39% annually, 23.1–24.8), and uterine cancer (0.35% annually, 45.5–48.7). In absolute terms, incident cases are projected to increase substantially due to population aging: breast cancer cases will rise by 76% (1.24–2.19 million cases), cervical cancer by 129% (288,000–660,000), ovarian cancer by 75% (183,000–320,000), and uterine cancer by 74% (360,000–627,000). Age-standardized mortality rates show divergent trends (Figure 8; Table S8).

Breast, cervical, and ovarian cancer mortality rates are projected to increase modestly (0.24%, 1.28%, and 0.12% annually, respectively), while uterine cancer mortality may decline slightly (−0.37% annually). However, absolute death counts will increase markedly across all cancers: breast cancer deaths are projected to rise by 78% (467,000–831,000), cervical by 113% (182,000–389,000), ovarian by 71% (149,000–255,000), and uterine by 61% (86,000–138,000). This discordance between stable or declining rates and rising absolute deaths reflects the dominant impact of population aging and growth. Overall disease burden measured by DALYs is projected to increase from 57% to 119% across all four cancers by 2040, driven primarily by mortality rather than morbidity (Figure S10; Table S8). Cervical cancer shows the largest relative increase (4.5–9.8 million DALYs, +119%), while absolute burden remains highest for breast cancer (10.8–18.6 million, +72%). Detailed year-by-year observed and predicted values, including 95% credible intervals and prediction errors for each cancer type and health outcome during the 2012–2021 validation period, are provided in Tables S9, S10, and S11.

### Model validation performance

Retrospective validation (1990–2011 training, 2012–2021 testing) demonstrated good predictive performance across all cancers (Tables S8, S9, S10, and S11). Mean absolute percentage errors were breast cancer 2.99%, cervical cancer 6.12%, ovarian cancer 6.25%, and uterine cancer 3.29%. R^2^ values ranged from 0.001 to 0.840 (mean, 0.252), with uterine cancer incidence showing the highest temporal stability (R^2^ = 0.840). All cancers exhibited systematic underestimation (negative mean bias), with actual burden growing 10%–15% faster than historical trends predicted, suggesting our 2040 projections may be conservative. Critically, for cervical cancer, the validation period (2012–2021) reflected populations born between 1957 and 1966 who entirely predated HPV vaccination programs (post-2006). The model’s good performance (mean absolute percentage error [MAPE], 6.12%) during this period validates its capacity to forecast trends in unvaccinated cohorts, while introducing substantial uncertainty for 2022–2040 projections when vaccinated cohorts begin entering menopausal ages.

### Causal association between age at menopause and breast cancer risk

MR analysis revealed associations between genetically predicted later age at menopause and increased breast cancer risk, with results from the primary dataset (ukb-b-17422) corroborated by the validation dataset (ieu-a-1004). In the primary analysis, genetically predicted later menopause was associated with increased overall breast cancer risk (inverse variance weighted [IVW]: OR, 1.24; 95% CI, 1.11–1.39; *p* < 0.001; 43 SNPs), with consistent direction across sensitivity methods. Subtype-specific analyses showed the strongest association for estrogen receptor (ER)+ breast cancer (IVW: OR, 1.28; 95% CI, 1.11–1.48; *p* < 0.001), where all five MR methods reached statistical significance. ER− breast cancer also showed a significant IVW estimate (OR, 1.26; 95% CI, 1.10–1.44; *p* < 0.001; 40 SNPs), partially supported by the weighted median (OR, 1.23; *p* = 0.034). HER2-negative breast cancer did not reach significance (IVW: OR, 1.12; 95% CI, 0.94–1.32; *p* = 0.204; 43 SNPs).

**Figure 9:**
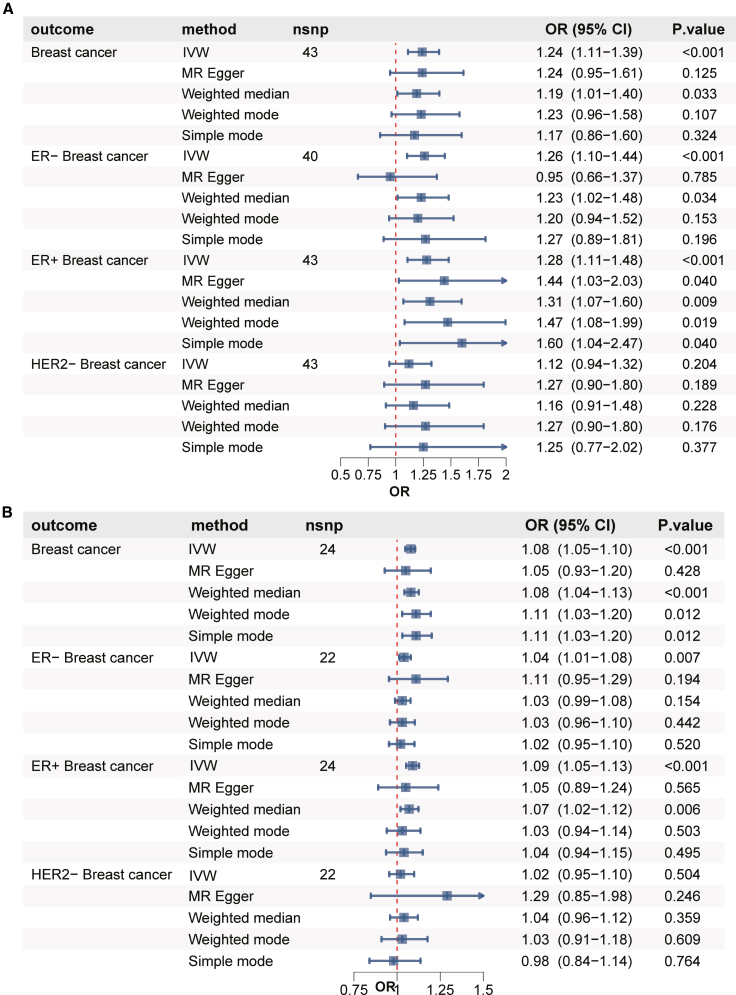
Mendelian randomization analysis of the causal association between age at menopause and risk of breast cancer Forest plots showing causal effect estimates of genetically predicted age at menopause on the risk of breast cancer and its molecular subtypes (overall breast cancer, ER−, ER+, and HER2− subtypes) using five MR methods. (A) Primary analysis using UK Biobank data. (B) Validation analysis using ReproGen consortium data.

The validation analysis confirmed these findings with attenuated but consistent effect sizes. Later menopause remained significantly associated with overall breast cancer (IVW: OR, 1.08; 95% CI, 1.05–1.10; *p* < 0.001; 24 SNPs) and ER+ breast cancer (IVW: OR, 1.09; 95% CI, 1.05–1.13; *p* < 0.001), with support from multiple sensitivity methods. ER− breast cancer showed a nominally significant IVW association (OR, 1.04; 95% CI, 1.01–1.08; *p* = 0.007), although sensitivity methods did not reach significance. HER2-negative breast cancer remained non-significant (IVW: OR, 1.02; *p* = 0.504). Overall, both datasets consistently support a causal role of later menopause in increasing overall and ER-positive breast cancer risk, with ER-positive subtypes demonstrating the most robust associations. The effect on ER-negative breast cancer was less consistently supported, and no significant association was observed for HER2-negative breast cancer (Figure 9).

### Causal association between age at menopause and gynecological cancer risk

**Figure 10:**
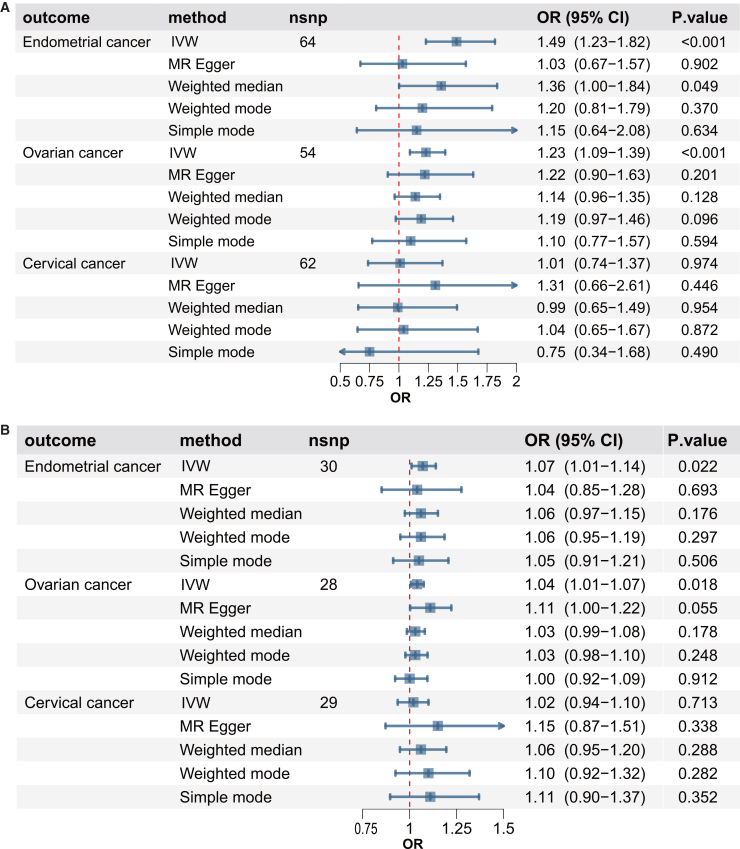
Mendelian randomization analysis of the causal association between age at menopause and risk of gynecological cancers Forest plots showing causal effect estimates of genetically predicted age at menopause on the risk of three gynecological cancers (endometrial cancer, ovarian cancer, and cervical cancer) using five MR methods. (A) Primary analysis using UK Biobank data. (B) Validation analysis using ReproGen consortium data.

**Figure 11:**
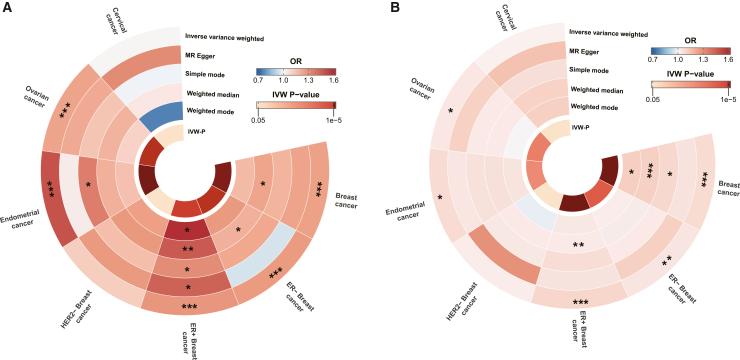
Circular heatmap of Mendelian randomization estimates for the causal association between age at menopause and gynecological and breast cancers Circular heatmap visualizing the causal effect estimates of genetically predicted age at menopause on the risk of seven cancer outcomes (overall breast cancer, ER− breast cancer, ER + breast cancer, HER2− breast cancer, endometrial cancer, ovarian cancer, and cervical cancer) using five MR methods. (A) Primary analysis using UK Biobank data. (B) Validation analysis using ReproGen consortium data. Color intensity represents the magnitude of odds ratios, with statistical significance indicated accordingly.

In the primary analysis, endometrial cancer showed the strongest association with later menopause (IVW: OR, 1.49; 95% CI, 1.23–1.82; *p* < 0.001), supported by the weighted median (OR, 1.36; 95% CI, 1.00–1.84, *p* = 0.049), although other sensitivity methods did not reach significance. Ovarian cancer also demonstrated a significant IVW estimate (OR = 1.23, *p* < 0.001), with consistent direction across sensitivity methods but none reaching statistical significance. Cervical cancer showed no causal association across all methods (IVW: OR, 1.01; 95% CI, 0.74–1.37; *p* = 0.974). The validation analysis confirmed the positive association for endometrial cancer (IVW: OR, 1.07; 95% CI, 1.01–1.14; *p* = 0.022) and ovarian cancer (IVW: OR, 1.04; 95% CI, 1.01–1.07; *p* = 0.018), albeit with attenuated effect sizes. MR-Egger for ovarian cancer showed a borderline estimate (OR, 1.11; 95% CI, 1.00–1.22; *p* = 0.055). Cervical cancer remained non-significant (IVW: OR, 1.02; 95% CI, 0.94–1.10; *p* = 0.713; 29 SNPs), consistent with the primary analysis (Figure 10). Overall, both datasets support a causal role of later menopause in increasing endometrial and ovarian cancer risk, while no evidence of a causal effect was observed for cervical cancer. Sensitivity analyses confirmed no evidence of pleiotropy or influential outliers for all cancer types (Figures S11–S24). Collectively, these findings provide robust genetic evidence supporting prolonged estrogen exposure through delayed menopause as a potential contributing factor to hormone-dependent cancers (breast, endometrial, and ovarian), while suggesting distinct etiological pathways for cervical cancer independent of reproductive hormone exposure (Figure 11).

## Discussion

This two-stage analysis provides crucial insights into gynecological and breast cancer burden among postmenopausal women aged ≥55 years and their causal relationship with menopausal age. Three key findings emerge. First, between 1990 and 2021, absolute cancer burden increased substantially worldwide, with divergent trends—breast and uterine cancer incidence rising (AAPC 0.17% and 0.47%), while cervical and ovarian cancers declining (AAPC, −0.75% and −0.38%). Second, profound socioeconomic inequalities persist, with cervical cancer concentrated in low-SDI regions (sub-Saharan Africa: >1,669 per 100,000 DALYs) versus breast and uterine cancers in high-SDI settings. Third, MR revealed associations between later menopause and increased risk of breast (OR, 1.24), endometrial (OR, 1.49), and ovarian cancers (OR, 1.23), but not cervical cancer (OR, 1.01; *p* > 0.05).

Our burden analysis reveals alarming inequalities against WHO’s 2030 cervical cancer elimination targets. In high-income countries, declining breast cancer mortality despite stable incidence (concentration index, from 0.11 to 0.01 for DALYs) exemplifies screening, treatment, and targeted therapy advances. Yet, uterine cancer incidence rises (AAPC 1.41%), driven by obesity and aging, with Black women experiencing 2%–3% annual increases. Conversely, sub-Saharan Africa faces increasing cervical cancer rates (AAPC 0.32%) despite global declines, with widening DALY inequalities (slope index, from −802.1 to −1,066.8), while breast cancer accelerates (AAPC 1.79%) amid limited infrastructure. Progress toward WHO’s 90-70-90 targets remains inadequate—only 21% of girls worldwide were vaccinated by 2022 and <5% of women in low-income countries were ever screened.^21^ This failure in translating knowledge into equity manifests in 94% of cervical cancer deaths occurring in low- and middle-income countries.

Modeling suggests achieving elimination could avert 74 million cases and prevent 62 million deaths by 2120, yet current trajectories show widening disparities.^22^ If 2022 rates persist, cases and deaths will increase 56.8% and 80.7% by 2050, concentrated in transitioning countries.^23^ Contributing factors include inadequate vaccination (<15% coverage in low-income countries), absent organized screening (visual inspection reaching <20% of eligible women), and treatment delays.^24^ The double burden in middle-income countries—declining cervical cancer alongside rising breast and uterine cancers—necessitates integrated responses. Countries in epidemiological transition cannot afford sequential approaches. Integrated platforms leveraging maternal-child health infrastructure for HPV vaccination, screening, and early detection represent cost-effective strategies requiring immediate implementation. Bayesian age-period-cohort (BAPC) projections reveal a looming escalation in gynecological cancer burden among postmenopausal women by 2040, driven primarily by demographic shifts rather than increasing risk. While age-standardized rates increase modestly (annual changes −0.4% to +1.8%), absolute case and death counts will surge 60%–129% across the four cancers, reflecting population aging and growth. The projected near doubling of deaths for breast (from 467,000 to 831,000) and cervical cancers (from 182,000 to 389,000) demands urgent healthcare system preparation. The current oncology capacity in many regions is already strained, and accommodating these increases requires substantial investments in screening, diagnostics, and treatment infrastructure. For cervical cancer—the only vaccine-preventable cancer analyzed—the steep projected rise (1.81% annually) underscores critical gaps in HPV vaccination and screening coverage, particularly in low-resource settings where 90% of cervical cancer deaths occur.

Women aged ≥55 during our validation period (2012–2021) were born between 1957 and 1966, entirely predating HPV vaccine availability (2006). These unvaccinated cohorts will continue dominating the ≥55 population through the early 2030s. Vaccinated cohorts (currently aged 12–30) will not substantially enter our analysis population until 2040–2050, beyond our projection horizon. Therefore, our 2022–2040 projections primarily reflect the natural history of unvaccinated cohorts aging into peak cancer incidence years, compounded by rapid population aging in low-SDI regions with minimal vaccination coverage (<15% in sub-Saharan Africa). The protective effects of current vaccination programs will materialize predominantly after 2040 when vaccinated cohorts reach menopausal ages. Validation results support this interpretation: the model accurately predicted unvaccinated cohort trends during 2012–2021 (MAPE, 6.12%), but the unprecedented mix of vaccinated and unvaccinated cohorts in future decades introduces uncertainty not captured by historical data. Our projections should, therefore, be interpreted as baseline scenarios reflecting continuation of historical trends, rather than comprehensive causal forecasts incorporating all potential future changes in modifiable risk factors. For uterine cancer specifically, the relatively modest projected increases warrant cautious interpretation given its strong obesity association. Historical stability may reflect countervailing protective factors including increased hysterectomy rates and hormonal contraceptive use, and actual future burden may exceed our projections if global obesity prevalence accelerates while protective factors plateau. Second, our projections are highly sensitive to evolving cancer screening technologies and practices that cannot be anticipated from historical data. If such technologies prove effective and achieve widespread implementation, particularly for ovarian cancer, which currently lacks effective screening modalities, actual future cancer burden could differ substantially from our projections in either direction—with earlier detection potentially increasing incidence while reducing mortality. Accordingly, our projections should be interpreted as contingent on the continuation of current screening paradigms.

Our MR findings align with and extend prior epidemiological evidence on menopause timing and breast cancer risk. The Collaborative Group meta-analysis of 117 studies reported a 1.029-fold increase in breast cancer risk per year of delayed menopause.^7^ Our MR analysis strengthens this association by establishing causality, mitigating confounding inherent in observational studies. Conversely, Allen-Brady et al. found that women with primary ovarian insufficiency (<40 years) had nearly twice the breast cancer risk (OR, 2.20) and a nominally significant increase in ovarian cancer risk (OR, 3.67).^25^ This counterintuitive finding—that both early and late menopause elevate breast cancer risk—suggests distinct etiological pathways. Prolonged estrogen exposure drives risk in later menopause, as supported by our MR data. In contrast, the Utah Population Database study implicates genetic variants, particularly in DNA damage response genes, which may impair ovarian function while increasing malignancy risk.^25^ These insights underscore the need for tailored screening and risk assessment strategies for women with varying menopause timing. For endometrial cancer, meta-analytic evidence demonstrates a dose-response relationship with relative risks of 1.04, 1.17, 1.57, and 2.08 for ages 47, 50, 54, and 57 years at menopause, with significance emerging when menopausal age exceeds 46.5 years.^10^ Our MR-derived estimate corroborates and supports this directionality. Notably, our null cervical cancer finding contradicts recent NHANES analyses reporting inverse associations. This discrepancy underscores MR’s value—observational findings likely reflect detection bias and healthcare access disparities rather than true biology. Notably, our null finding for cervical cancer directly contradicts recent NHANES analyses reporting inverse associations between menopausal age and cervical cancer risk in postmenopausal women.^15^^,^^16^ Given HPV infection drives cervical carcinogenesis,^26^ our genetic evidence suggests reproductive hormone exposure plays no independent role, which is consistent with previous studies.^27^

The causal associations illuminate hormone-dependent carcinogenesis fundamentals. Later menopause extends endogenous estrogen and progesterone exposure, driving proliferation in hormone-responsive breast and endometrial tissues through ER pathways.^10^ Prolonged cyclical stimulation increases cellular divisions, amplifying oncogenic mutation opportunities.^28^ In breast tissue, estrogen promotes epithelial proliferation while inhibiting apoptosis—particularly evident in our subtype analyses showing stronger ER-positive versus ER-negative effects. This heterogeneity suggests hormone-independent pathways contribute to ER-negative cancer, although endocrine exposure remains contributory across subtypes. The relationship between exogenous hormone exposure and breast cancer risk is nuanced and regimen dependent. Contemporary 2025 evidence confirms that combined estrogen-progestogen therapy elevates breast cancer risk, with norethisterone-estradiol showing the highest risk and dydrogesterone-estradiol the lowest among combination regimens.^29^ However, randomized clinical trial evidence from the Women’s Health Initiative (WHI) conjugated equine estrogen (CEE)-alone trial demonstrated that estrogen-alone therapy was associated with reduced breast cancer incidence and mortality during long-term follow-up,^30^ a finding subsequently corroborated by meta-analyses of smaller randomized trials.^31^ This critical distinction suggests that the progestogen component—rather than estrogen alone—primarily drives excess breast cancer risk in combined HRT users. Our MR findings, which reflect prolonged endogenous estrogen and progesterone exposure through delayed menopause, may align more closely with the combined hormonal milieu of the perimenopausal transition, although further mechanistic studies are warranted. For endometrial cancer, unopposed estrogen stimulation—from perimenopausal anovulatory cycles and postmenopausal peripheral aromatization—drives hyperplasia progression to adenocarcinoma.^32^ Our strong causal estimate reflects this direct mitogenic effect, consistent with established obesity links (increased adipose aromatase), estrogen-only HRT, and endometrial cancer risk.^33^^,^^34^^,^^35^ The modest ovarian cancer association reflects biological complexity. Emerging evidence shows estrogen increases tumor cell proliferation through ERα, while ERβ exerts antiproliferative effects, with ERβ loss during progression representing important tumorigenesis.^36^ Our findings may reflect the “incessant ovulationˮ hypothesis—later menopause means more lifetime ovulatory cycles, and repetitive ovulatory trauma with inflammatory repair could promote surface epithelium transformation.^37^^,^^38^ Importantly, exogenous hormone exposure further substantiates a role for estrogen in ovarian carcinogenesis: long-term follow-up of the WHI randomized trials demonstrated that estrogen-alone therapy (CEE) was associated with increased ovarian cancer incidence.^39^ Cervical cancer’s distinct HPV-driven etiology explains our null finding. Viral oncoproteins E6 and E7 inactivate *p53* and *Rb* independently of reproductive hormone exposure duration.^40^^,^^41^ Our genetic evidence definitively clarifies that observational early menopause-cervical cancer associations reflect socioeconomic confounding affecting both menopausal timing and screening access. Our causal findings inform risk stratification and precision prevention. Women with genetically later menopause represent higher-risk populations warranting enhanced surveillance. Recent MR-phenome-wide studies confirm later menopause increases breast, endometrial, and ovarian cancer risks while reducing osteoporosis, fracture, and type 2 diabetes risks—highlighting trade-offs requiring individualized assessment.^42^ Polygenic risk scores incorporating menopausal timing variants could refine screening recommendations.^43^ However, clinical translation requires diverse ancestry validation, as current data derive overwhelmingly from European populations.

The apparent contradiction between our MR findings and recent NHANES-based observational studies warrants careful interpretation within a biological versus sociological confounding framework. Our MR analysis, by using genetic variants as instruments immune to conventional confounding, captures the potential biological effect of prolonged endogenous estrogen exposure—indexed by later natural menopause—on hormone-dependent cancer risk. In contrast, the inverse associations reported in observational studies, where earlier menopause appears paradoxically associated with higher cervical, ovarian, and uterine cancer risk, likely reflect sociological confounding pathways rather than true biological effects.

A plausible confounding mechanism is as follows: women of lower socioeconomic status may simultaneously experience (1) earlier natural menopause, driven by higher allostatic stress burden, poorer nutritional status, higher smoking prevalence, greater parity, and limited access to healthcare, and (2) higher observed cancer rates, attributable not to menopause timing per se, but to lower rates of cancer screening, later stage at diagnosis, reduced access to effective treatment, and greater exposure to oncogenic risk factors including persistent HPV infection. This socioeconomic confounding creates a spurious statistical association between earlier menopause and higher cancer risk in observational data, which does not reflect a biological causal pathway. The MR approach, by anchoring analysis to genetic determinants of menopause timing that are allocated at conception and independent of socioeconomic position, effectively breaks this confounding chain and reveals the underlying biological relationship. This comparison underscores a core strength of MR in disentangling biological from sociological associations—particularly important in global health contexts where menopausal timing, cancer screening access, and socioeconomic conditions are deeply intertwined.

In conclusion, this study integrates global epidemiological surveillance with MR to provide comprehensive insights into gynecological and breast cancer burden among postmenopausal women. Our findings reveal profound and widening health inequalities, with preventable cervical cancer mortality concentrated in the poorest regions while hormone-dependent malignancies rise in transitioning countries. By identifying genetic associations between later menopause and increased risk of breast, endometrial, and ovarian cancers through prolonged estrogen exposure, while confirming no effect on cervical cancer, we clarify distinct biological pathways underlying these malignancies. As the global population of older women approaches unprecedented scale, the central challenge is one of justice and political will—scientific knowledge and effective interventions exist to prevent the vast majority of these cancers, yet widening inequalities persist. Translating scientific progress into equitable outcomes requires not only technical solutions but also sustained commitment to ensure all women benefit from advances in cancer prevention and treatment, regardless of their circumstances.

### Limitations of the study

Our principal strength lies in integrating comprehensive epidemiological surveillance with causal inference. GBD 2021 provides standardized metrics across 204 countries spanning three decades, and focusing specifically on women aged ≥55 years captures the postmenopausal population facing maximal risk—a subgroup systematically obscured by conventional all-age standardized analyses. Our MR approach employs five complementary estimation methods, rigorous pleiotropy exclusion including outcome-specific SNP sets, and independent dataset validation using the ReproGen consortium, providing robustness beyond what single-method analyses can offer.

Several limitations warrant careful consideration. Regarding the GBD data, estimates rely on complex modeling with heterogeneous source quality. Cancer registration completeness differs markedly—near-universal in high-income settings but fragmentary in low-SDI countries—potentially underestimating burden precisely where it is the greatest. This measurement paradox is particularly acute for cervical cancer, where over 94% of deaths occur in low- and middle-income countries that contribute least to surveillance infrastructure.

Regarding the BAPC projections, it should first be noted that the BAPC framework is a trend extrapolation approach that does not incorporate external covariates such as obesity prevalence, screening coverage, or treatment advances as explicit predictors; rather, the influence of such factors is implicitly captured within the historical age-period-cohort trends observed during 1990–2021, with uncertainty quantified using posterior distributions. Against this background, three specific caveats apply. First, all projections assume continuation of historical trends and cannot capture abrupt changes from novel therapies, policy shifts, or unforeseen events, with the 19-year projection horizon introducing increasing uncertainty reflected in widening prediction intervals. Second, HPV vaccination—introduced in 2006 and increasingly adopted globally—may substantially alter cervical cancer trajectories, but vaccinated cohorts will not substantially enter the ≥55 population until after 2040, beyond our projection horizon; our projections therefore reflect the natural history of unvaccinated cohorts and should be interpreted as demographic-driven baseline scenarios rather than definitive forecasts. Third, for hormone-dependent cancers, therapeutic advances may improve survival in ways not captured by historical trends, and for uterine cancer specifically, projections may be conservative if global obesity prevalence accelerates beyond historical rates while protective factors such as hormonal contraceptive use plateau.

Regarding the MR analysis, two fundamental interpretive boundaries must be acknowledged. First, our MR findings pertain specifically to the effects of genetically determined age at natural menopause and cannot be directly translated into clinical recommendations regarding interventions that modify menopausal timing. Genetic variants associated with later natural menopause primarily reflect biological pathways governing ovarian reserve and follicular atresia—mechanisms distinct from those involved in surgical oophorectomy, exogenous hormone administration, or pharmacological ovarian suppression—and direct extrapolation to guide clinical decision-making would be methodologically inappropriate. Second, all genetic instrumental variables and outcome genome-wide association study (GWAS) data were derived exclusively from populations of European ancestry, creating a critical disconnect between the global scope of our burden analysis and the geographic applicability of our causal inferences. Genetic architecture differs across ancestries, such that our instrumental variables may have reduced strength in non-European contexts due to differences in linkage disequilibrium patterns and allele frequencies. Furthermore, gene-environment interactions may modify causal effect magnitudes across populations with different HPV prevalence, obesity trajectories, and hormonal profiles. This limitation is particularly consequential for cervical cancer, where our null MR finding was interpreted as reflecting HPV-driven etiology, but requires validation in non-European populations where HPV subtype distributions and immunogenetic factors may differ. We explicitly caution that our causal conclusions currently apply primarily to European ancestry populations, and generalizability to South Asian, East Asian, sub-Saharan African, and Latin American populations—who bear the greatest gynecological cancer burden—requires dedicated multi-ancestry replication studies leveraging emerging resources such as the H3Africa Consortium and the China Kadoorie Biobank.

## Resource availability

### Lead contact

Further information and requests for resources should be directed to and will be fulfilled by the lead contact, Tao Zeng (zengt56@mail2.sysu.edu.cn).

### Materials availability

This study did not generate new unique reagents.

### Data and code availability

Data: The data used in this study are available at Open Science Framework: https://doi.org/10.17605/OSF.IO/SBQ25. The URL and DOI for the repository are also listed in the key resources table. Code: All original codes used in this study are available at Open Science Framework: https://doi.org/10.17605/OSF.IO/SBQ25, https://doi.org/10.17605/OSF.IO/SBQ25. The URL and DOI for the repository are also listed in the key resources table. Any additional information required to reanalyze the data reported in this paper is available from the lead contact upon request.

## Acknowledgments

The authors gratefully acknowledge the Global Burden of Disease study and the IEU OpenGWAS project for providing the data essential to this research. We also extend our appreciation to the researchers and funding bodies involved in curating and maintaining these valuable public resources. Funding: this research was funded by the 10.13039/501100001809National Natural Science Foundation of China (grant no. 82404010) and the 10.13039/501100018542Natural Science Foundation of Sichuan Province, China (grant no. 2025ZNSFSC1696).

## Author contributions

S.L., investigation, data curation, funding acquisition, and writing – original draft. Y.T., methodology, investigation, writing – original draft, and funding acquisition. C.C., investigation and data curation. D.D., formal analysis, validation, and visualization. J.Y., methodology, formal analysis, and validation. T.Z., conceptualization, supervision, project administration, and writing – review and editing.

## Declaration of interests

The authors declare that they have no competing interests.

## STAR★Methods

### Key resources table

**Table:** 

REAGENT or RESOURCE	SOURCE	IDENTIFIER
**Software and algorithms**		

R programming language 4.3.0	R Foundation, USA	https://www.r-project.org
RStudio 2024.04.2	Posit, USA	https://posit.co/downloads/
Joinpoint Regression Program 5.1.0.0	National Cancer Institute, USA	https://surveillance.cancer.gov/joinpoint/

**Other**		

Source data (GBD)	Global Health Data Exchange	https://vizhub.healthdata.org/gbd-results/
Source data (GWAS)	IEU Open GWAS Project	https://opengwas.io/datasets/
Analysis code and processed data	Open Science Framework	URL: https://doi.org/10.17605/OSF.IO/SBQ25 https://doi.org/10.17605/OSF.IO/SBQ25

### Experimental model and study participant details

This study did not generate experimental model or enroll subjects. All data used in this study were obtained from the the Global Burden of Disease (GBD) database and IEU Open GWAS Project.

### Method detailsdetails

#### Data source and framework

##### Global Burden of Diseases (GBD) data source

This study utilized data from the GBD 2021, a comprehensive epidemiological effort that systematically quantifies health impacts across 371 diseases and injuries in 204 countries and territories. Women aged 55 years and older were selected as a representative fully postmenopausal population, consistent with prior GBD subgroup analyses employing this age threshold.^44^^,^^45^ This cutoff was chosen to capture the postmenopausal population while accommodating known variability in natural menopausal age across global regions. For female-specific cancers (breast, cervical, ovarian, and uterine), we extracted absolute counts and rates (per 100,000 population) for prevalence, incidence, mortality, and DALYs from 1990 to 2021, using the GBD online visualization tool (https://vizhub.healthdata.org/gbd-results/). Disease definitions followed the International Classification of Diseases (ICD) coding system (Table S12).

Regional development levels were assessed using the Socio-Demographic Index (SDI), a composite measure of income per capita, educational attainment, and fertility rate (Table S13). The SDI is a composite measure, scaled from 0 to 1, that serves as a geometric mean of the lag-distributed income per capita, the average educational attainment of the population aged 15 and above, and the total fertility rate for women under 25.^46^ Based on similarities in socioeconomic, demographic, and health system characteristics, the GBD study classifies 204 countries and territories into seven super-regions—High-income, Latin America and Caribbean, Sub-Saharan Africa, North Africa and Middle East, South Asia, Southeast Asia, East Asia, and Oceania, and Central Europe, Eastern Europe, and Central Asia—to facilitate more scientific analysis and comparison of disease burden patterns across regions with different development levels.

#### MR data sources

##### Genetic instrumental variables for age at menopause

Genetic instrumental variables for age at menopause were derived from two independent GWAS of European ancestry populations. The primary exposure dataset was obtained from the UK Biobank (GWAS ID: ukb-b-17422), comprising 143,819 women and 9,851,867 SNPs. A validation dataset from the ReproGen consortium (GWAS ID: ieu-a-1004), including 69,360 women and 26,414,677 SNPs, was used to replicate the causal estimates (Table S14). SNPs associated with age at menopause at genome-wide significance (*p* < 5 × 10^−8^) were selected as instrumental variables, and linkage disequilibrium clumping (r^2^ < 0.001, window = 10,000 kb) was applied to ensure independence.

##### Outcome data for gynecological and breast cancers

GWAS data for these four cancers were retrieved from large-scale European-ancestry cohorts (Table S14). Breast cancer data included 33,832 individuals^47^(15,748/18,084 cases/controls, 13,011,123 SNPs). Specifically, for breast cancer, we utilized summary statistics for its major molecular subtypes to investigate heterogeneity in causal effects: ER-breast cancer^48^ (127,442 individuals, 21,468 cases), ER + breast cancer^47^ (22,286 individuals, 4,202 cases), and HER2-breast cancer (123,579 individuals, 7,355 cases). Endometrial cancer data comprised 240,027 individuals^49^ (12,906 cases). Ovarian cancer included 66,450 individuals^27^ (25,509 cases), and cervical cancer encompassed 239,158 individuals^49^ (909 cases).

#### Statistical analysis

##### Joinpoint regression analysis

Temporal trends in gynecological and breast cancers burden among women aged 55 years and older were evaluated using Joinpoint Regression Program (Version 5.1.0.0, National Cancer Institute). This method fits segmented log-linear regression models to identify statistically significant inflection points in time series data while optimizing model parsimony. The Grid Search method was applied with minimum 2 observations from a joinpoint to either data end and between consecutive joinpoints, with 0 additional grid points. Joinpoint numbers ranged from 0 to 6, selected via Permutation Test (4,499 permutations, α = 0.05). The AAPC segment spanned 1990–2021. Confidence intervals were calculated using the parametric method with 5,001 resamples. Annual percentage change (APC) with 95% CIs was estimated for each segment based on incidence, prevalence, mortality, and DALY rates. Average annual percentage change (AAPC), calculated as the time-weighted geometric mean of APCs, summarized overall trends across the study period.

##### Correlation analysis between SDI and cancer burden

To examine associations between socioeconomic development and gynecological and breast cancers burden at the country level, we performed correlation analyses between SDI values and rates (incidence, mortality, prevalence, and DALYs) for each cancer type in 2021.Both Pearson (r) and Spearman (ρ) correlation coefficients were calculated to assess linear and monotonic relationships. To evaluate non-linearity, we compared three models: (1) linear regression (y = β_0_ + β_1_x), (2) quadratic regression (y = β_0_ + β_1_x + β_2_x^2^), and (3) LOESS smoothing (span = 0.75). Coefficient of determination (R^2^) quantified variance explained by each model. Non-linearity was determined if the quadratic term was significant (*p* < 0.05, ANOVA) with ΔR^2^ > 0.05 versus linear model. Scatterplots displayed LOESS curves (overall trend) with dashed quadratic curves added for non-linear relationships.

##### Assessment of socioeconomic inequalities

Socioeconomic disparities in gynecological and breast cancer burden were quantified using the slope index of inequality (SII) and the concentration index (CI), two complementary measures recommended for assessing health inequalities across socioeconomic gradients.^50^^,^^51^ The CI is a relative measure derived from the concentration curve, which plots the cumulative proportion of the population ranked by SDI against the cumulative proportion of cancer burden. The CI ranges from −1 to +1, with zero indicating no inequality; positive values indicate burden concentrated among higher-SDI populations and negative values among lower-SDI populations. The SII is an absolute measure calculated by regressing cancer burden indicators against relative socioeconomic position (midpoint of cumulative population distribution ranked by SDI), using weighted least squares to account for heteroskedasticity. The SII represents the absolute rate difference (per 100,000) between the lowest and highest extremes of the SDI distribution. Together, the CI captures relative distribution of burden across the socioeconomic spectrum, while the SII quantifies the absolute rate difference.

##### Future trend projections using Bayesian Age-Period-Cohort models

We projected future trends in incidence, mortality, and DALYs for breast, cervical, ovarian, and uterine cancers among postmenopausal women (aged ≥55 years) from 2022 to 2040 using Bayesian Age-Period-Cohort (BAPC) models. BAPC models were employed to project cancer burden through 2040, implemented using the BAPC package (version 0.0.36) in R with Integrated Nested Laplace Approximations (INLA) for Bayesian inference. The BAPC model decomposes observed cancer rates into three temporal components: age effects (αi) capturing biological relationships between aging and cancer risk; period effects (βj) representing factors simultaneously affecting all age groups at a given calendar time such as diagnostic practices, screening implementation, and treatment advances; and cohort effects (γk) capturing exposures specific to individuals born in the same time period, reflecting generational differences in lifestyle and environmental exposures.^52^^,^^53^ The model assumes that the logarithm of expected rates follows an additive structure: log(λij) = μ + αi + βj + γk, where λij is the expected rate for age group i in period j, μ is the intercept, and k indexes birth cohort. Second-order random walk (RW2) priors were applied to all temporal effects, assuming independent mean-zero normal distributions on second differences to ensure smooth trends while penalizing deviations from linearity. For projections, period and cohort effects were extrapolated linearly following the RW2 structure with cubically increasing variance, reflecting growing uncertainty over longer projection horizons. Population projections from the UN Population Division (2020 medium-variant) were used as denominators to convert projected rates to absolute case numbers and to account for demographic changes in population size and age structure, rather than as predictors within the BAPC model. The BAPC framework is a trend extrapolation approach that does not incorporate external covariates (such as obesity prevalence, screening coverage, or treatment advances) as explicit predictors; rather, the influence of such factors is implicitly captured within the historical age-period-cohort trends observed during 1990–2021. Uncertainty was quantified using posterior distributions, with 50%, 80%, 95%, and 99% uncertainty intervals reported.

For each cancer-measure combination, we fitted BAPC models to global historical data (1990–2021). The generic function parameter was set to 5 to balance model flexibility and projection stability. Projections were generated with full posterior distributions, reporting point estimates (posterior means) and multiple uncertainty intervals (UI): 80% and 95%. All rates were age-standardized using WHO world standard population weights. Model validation was performed through retrospective testing, fitting models to 1990–2011 data and comparing 2012–2021 predictions against observed values.

#### MR analysis

##### Instrumental variable selection

Genetic variants associated with age at natural menopause were selected as instrumental variables (IVs) following stringent criteria to satisfy the core MR assumptions. SNPs were selected based on genome-wide significance (*p* < 5 × 10^−8^) and clumped for linkage disequilibrium (r^2^ < 0.001, clumping window = 10,000 kb) using the 1000 Genomes European reference panel.^54^ Palindromic SNPs with ambiguous strand orientation and SNPs with incompatible alleles were excluded during harmonization. Instrument strength was assessed using F-statistics, with all SNPs demonstrating F > 10, indicating minimal weak instrument bias.^55^^,^^56^

To minimize potential pleiotropy and satisfy the core assumptions of Mendelian randomization, we performed systematic exclusion of SNPs associated with the outcomes or key confounders. All candidate SNPs were evaluated using trait annotations from the GWAS Catalog via the LDlink platform (https://ldlink.nih.gov/), and SNPs showing genome-wide significant associations (*p* < 5 × 10^−8^) with gynecological conditions (endometriosis, polycystic ovary syndrome, uterine fibroids) or reproductive timing factors (age at menarche, age at first birth) were excluded. Separate instrumental variable sets were constructed for each cancer outcome after removing outcome-specific and confounder-related SNPs. For the primary dataset (ukb-b-17422), this yielded 59 SNPs for breast cancer, 82 for cervical cancer, 82 for endometrial cancer, and 72 for ovarian cancer (Table S15). The same filtering procedure was applied to the validation dataset (ieu-a-1004), and the corresponding instrumental variable sets are provided in Table S16.

##### MR estimation methods

We employed five complementary MR methods to triangulate causal estimates^57^^,^^58^^,^^59^^,^^60^: (1) IVW method served as the primary analysis, providing a weighted average of Wald ratio estimates under multiplicative random-effects meta-analysis; (2) Weighted median method, which remains consistent when up to 50% of information comes from invalid instruments; (3) MR-Egger regression, allowing for directional pleiotropy with the intercept testing for average horizontal pleiotropy; (4) Weighted mode and (5) Simple mode methods, which identify the most common causal estimate across SNP clusters. Causal estimates were reported as odds ratios (ORs) with 95% confidence intervals (CIs) per 1-standard deviation (SD) increase in age at menopause, with statistical significance defined as *p* < 0.05 for the primary IVW analysis. Multiple sensitivity analyses evaluated the validity of MR assumptions: MR-Egger intercept test detected directional pleiotropy (*p* < 0.05 indicates pleiotropy). Leave-one-out analysis identified influential SNPs by iteratively excluding each SNP and recalculating the IVW estimate. Scatterplots displayed SNP-specific exposure-outcome associations alongside causal estimates from each MR method to visualize consistency; Funnel plots assessed potential pleiotropy through asymmetry inspection.

##### Subgroup and validation analyses

Subgroup MR analyses were performed stratified by breast cancer molecular subtypes (ER+, ER-, HER2-) to investigate etiological heterogeneity. Validation analyses using independent GWAS datasets tested the reproducibility of primary findings. All analyses were performed using the TwoSampleMR package (version 0.6.29) in R software (version 4.3.0), with two-sided *p* < 0.05 considered statistically significant.

### Quantification and statistical analysis

Data processing and analysis were performed using R software (version 4.3.0; R Foundation, USA), RStudio (version 2024.04.2; Posit, USA), and Zstats v1.0 (www.zstats.net). The study focused on women aged ≥55 years across 204 countries and territories from 1990 to 2021. All estimates (incidence, prevalence, mortality, and DALYs) are presented with 95% uncertainty intervals (UIs) derived from the GBD 2021 model. Temporal trends in age-standardized rates were quantified using Joinpoint Regression Program (version 5.1.0.0; National Cancer Institute, USA), with annual percentage change (APC) and average annual percentage change (AAPC) reported alongside 95% confidence intervals (CIs); an AAPC >0 indicates an increasing trend and <0 a decreasing trend. Detailed APC estimates by year, cancer type, and region are presented in the figure legends, Results section, and Tables S1, S2, S3, S4, and S5. Associations between Socio-Demographic Index (SDI) and cancer burden rates across 204 countries were assessed using Pearson (r) and Spearman (ρ) correlation coefficients, with linear, quadratic, and LOESS regression models compared by R^2^; results are reported in the Results section and Table S6. Socioeconomic inequalities were quantified using the Concentration Index and Slope Index of Inequality (SII), with values for 1990 and 2021 detailed in Table S7 and Figures 6, S7, S8, S9. Future burden projections (2022–2040) were generated using Bayesian Age-Period-Cohort (BAPC) models implemented via the BAPC R package (version 0.0.36) with INLA, reporting posterior means with 80% and 95% uncertainty intervals; model validation metrics (MAPE, R^2^) and year-by-year predicted values are provided in Tables S8, S9, S10, S11, S12, S13, S14, S15, and S16. Mendelian randomization analyses were conducted using the TwoSampleMR R package (version 0.6.29), with causal estimates from five complementary methods (IVW, weighted median, MR-Egger, weighted mode, simple mode) expressed as odds ratios (ORs) with 95% CIs per 1-SD increase in genetically predicted age at menopause; the exact number of SNPs per instrumental variable set, sensitivity analysis results, and per-method estimates are detailed in the Results section, Figures 9, 10, 11, S11–S24, and Tables S15 and S16. Statistical significance was defined as two-sided *p* < 0.05 throughout.

## Supplemental information

Document S1. Figures S1–S24

Table S1. Incidence of gynecological and breast cancers in postmenopausal women aged ≥55 years at the global level and across different regions in 1990 and 2021, and changes over this period

Table S2. Prevalence of gynecological and breast cancers in postmenopausal women aged ≥55 years at the global level and across different regions in 1990 and 2021, and changes over this period

Table S3. APC in incidence, prevalence, mortality, and DALY rates of gynecological and breast cancers at the global level and across different regions

Table S4. DALYs of gynecological and breast cancers in postmenopausal women aged ≥55 years at the global level and across different regions in 1990 and 2021, and changes over this period

Table S5. Mortality of gynecological and breast cancers in postmenopausal women aged ≥55 years at the global level and across different regions in 1990 and 2021, and changes over this period

Table S6. Association between SDI and gynecological and breast cancer burden metrics across 204 countries and territories in 2021

Table S7. Concentration index and SII for gynecological and breast cancer burden metrics stratified by sociodemographic index in 1990 and 2021

Table S8. Summary of projected trends in incidence, mortality, and DALYs for gynecological and breast cancers among postmenopausal women aged ≥55 years from 2022 to 2040

Table S9. Retrospective validation results for age-standardized DALY rates of four gynecological and breast cancers during the 2012–2021 testing period

Table S10. Retrospective validation results for age-standardized mortality rates of four gynecological and breast cancers during the 2012–2021 testing period

Table S11. Retrospective validation results for age-standardized incidence rates of four gynecological and breast cancers during the 2012–2021 testing period

Table S12. ICD-10 codes used to define gynecological and breast cancers in the GBD 2021 framework

Table S13. SDI values for 204 countries and territories as defined in GBD 2021

Table S14. Summary of GWAS datasets used for exposure and outcome variables in the primary and validation Mendelian randomization analyses

Table S15. Genetic instrumental variables for age at natural menopause derived from the UK Biobank GWAS (ukb-b-17422) used in the primary Mendelian randomization analysis

Table S16. Genetic instrumental variables for age at natural menopause derived from the ReproGen consortium GWAS (ieu-a-1004) used in the validation Mendelian randomization analysis
