# Supplementary material for: Global burden trends and menopause timing associations for gynecological and breast cancers in postmenopausal women
Source: iScience. 2026 Jun 1;29(6):115967. doi: 10.1016/j.isci.2026.115967 (PMC13253087; doi:10.1016/j.isci.2026.115967)

iScience, Volume *29*

Supplemental information

Global burden trends and menopause timing associations for gynecological and breast cancers in postmenopausal women

Shuxin Li, Yu Tian, Cui Chen, Dengyi Duan, Jinhua Yan, and Tao Zeng

Figure S1. Global trends in prevalence of gynecological and breast cancers among postmenopausal women aged ≥55 years. Annual incident cases and incidence rates for (A) breast cancer, (B) cervical cancer, (C) ovarian cancer, and (D) uterine cancer from 1990 to 2021.

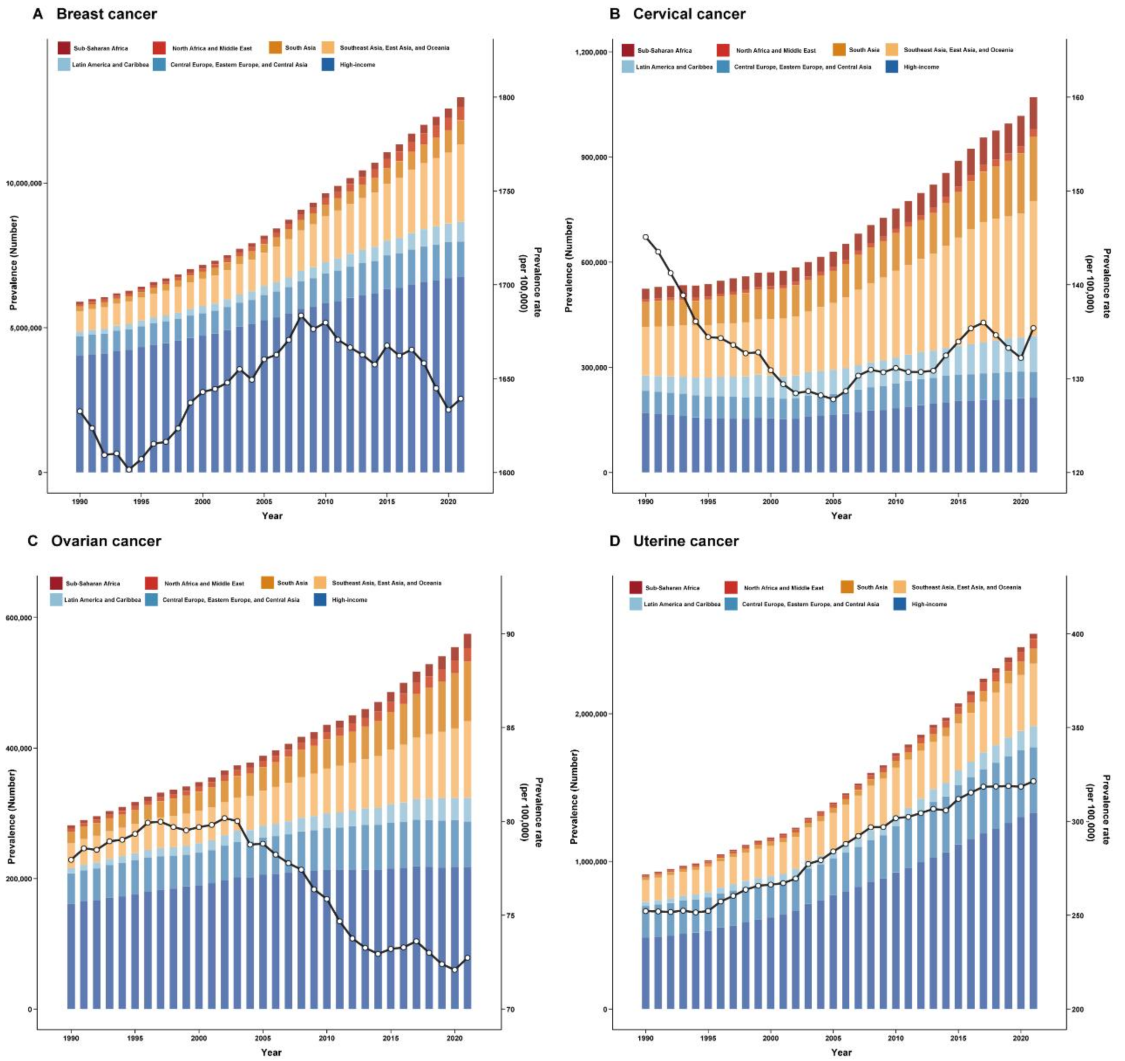

Figure S2. Global trends in death of gynecological and breast cancers among postmenopausal women aged ≥55 years. Annual incident cases and incidence rates for (A) breast cancer, (B) cervical cancer, (C) ovarian cancer, and (D) uterine cancer from 1990 to 2021.

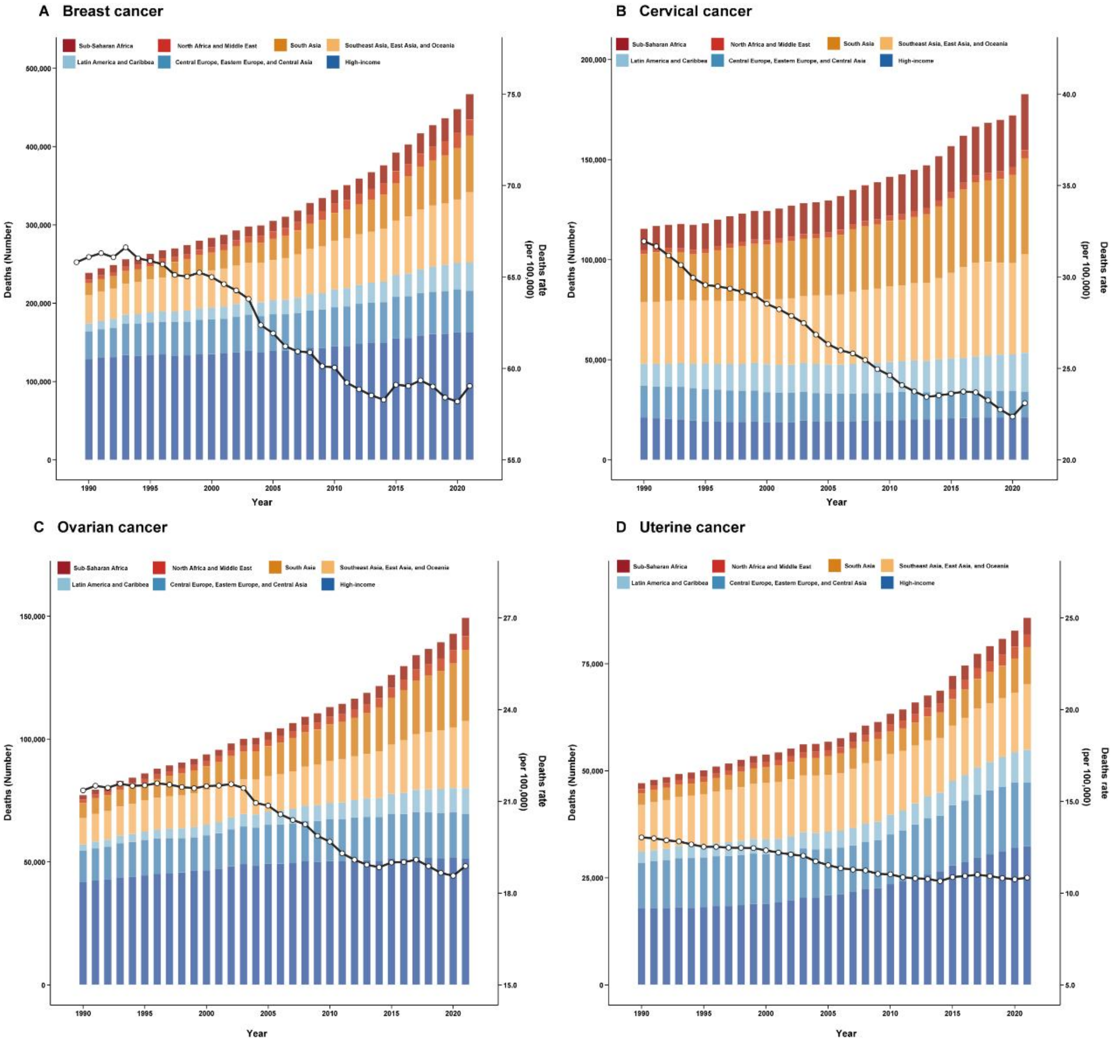

Figure S3. Regional trends in prevalence rates of gynecological and breast cancers among postmenopausal women aged ≥55 years. A-D: Prevalence rates (per 1000,000 women aged ≥55 years) for breast cancer **(A)**, cervical cancer**(B)**, ovarian cancer **(C)**, and uterine cancer **(D)** across seven global regions from 1990 to 2021. **E:** Heatmaps displaying the average annual percentage change (AAPC) in prevalence rates for breast cancer, cervical cancer, uterine cancer, and ovarian cancer across different global regions from 1990 to 2021.

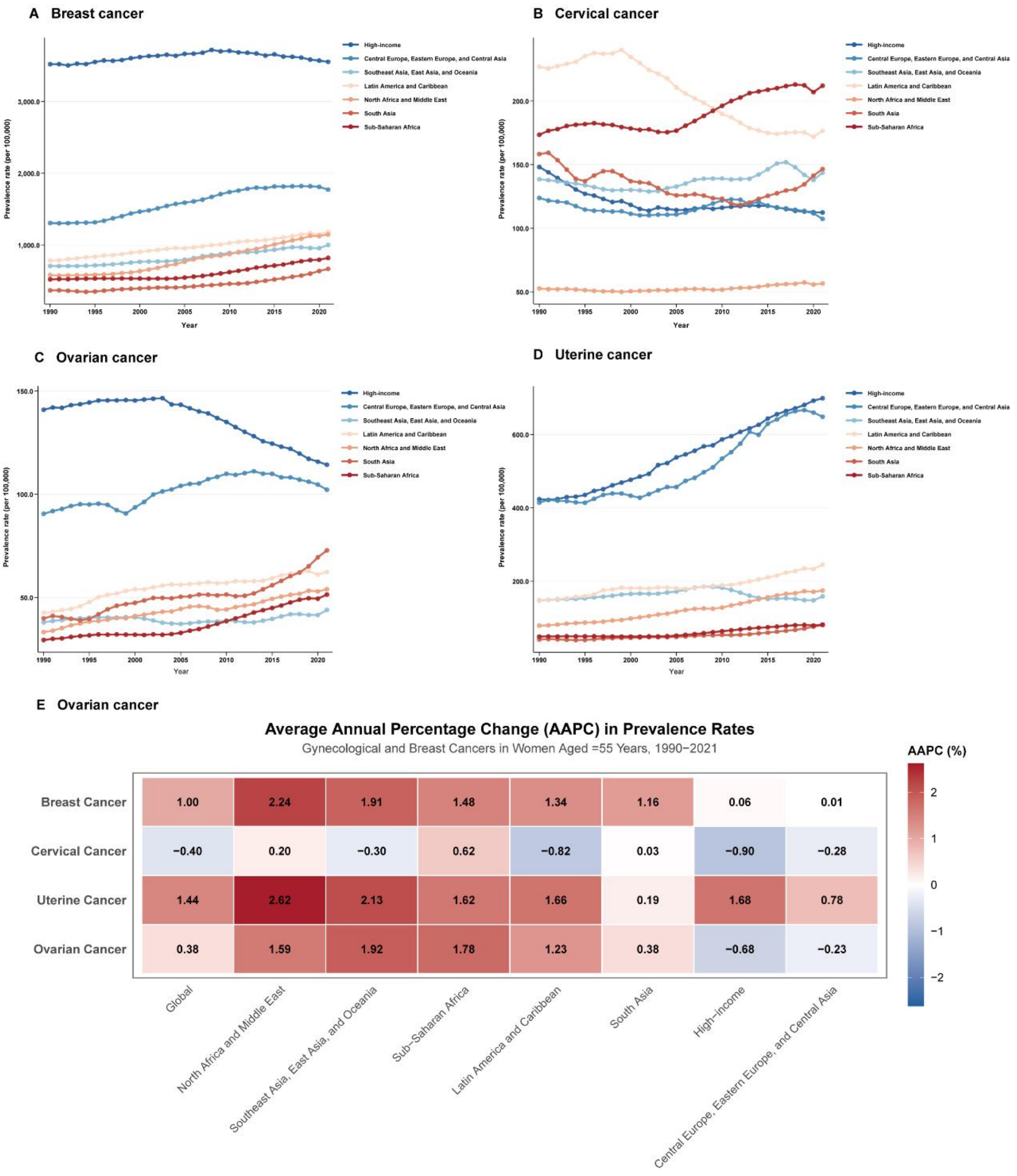

Figure S4. Regional trends in death rates of gynecological and breast cancers among postmenopausal women aged ≥55 years. A-D: Death rates (per 1000,000 women aged ≥55 years) for (A) breast cancer, (B) cervical cancer, (C) ovarian cancer, and (D) uterine cancer across seven global regions from 1990 to 2021. **E:** Heatmaps displaying the average annual percentage change (AAPC) in mortality rates for breast cancer, cervical cancer, uterine cancer, and ovarian cancer across different global regions from 1990 to 2021.

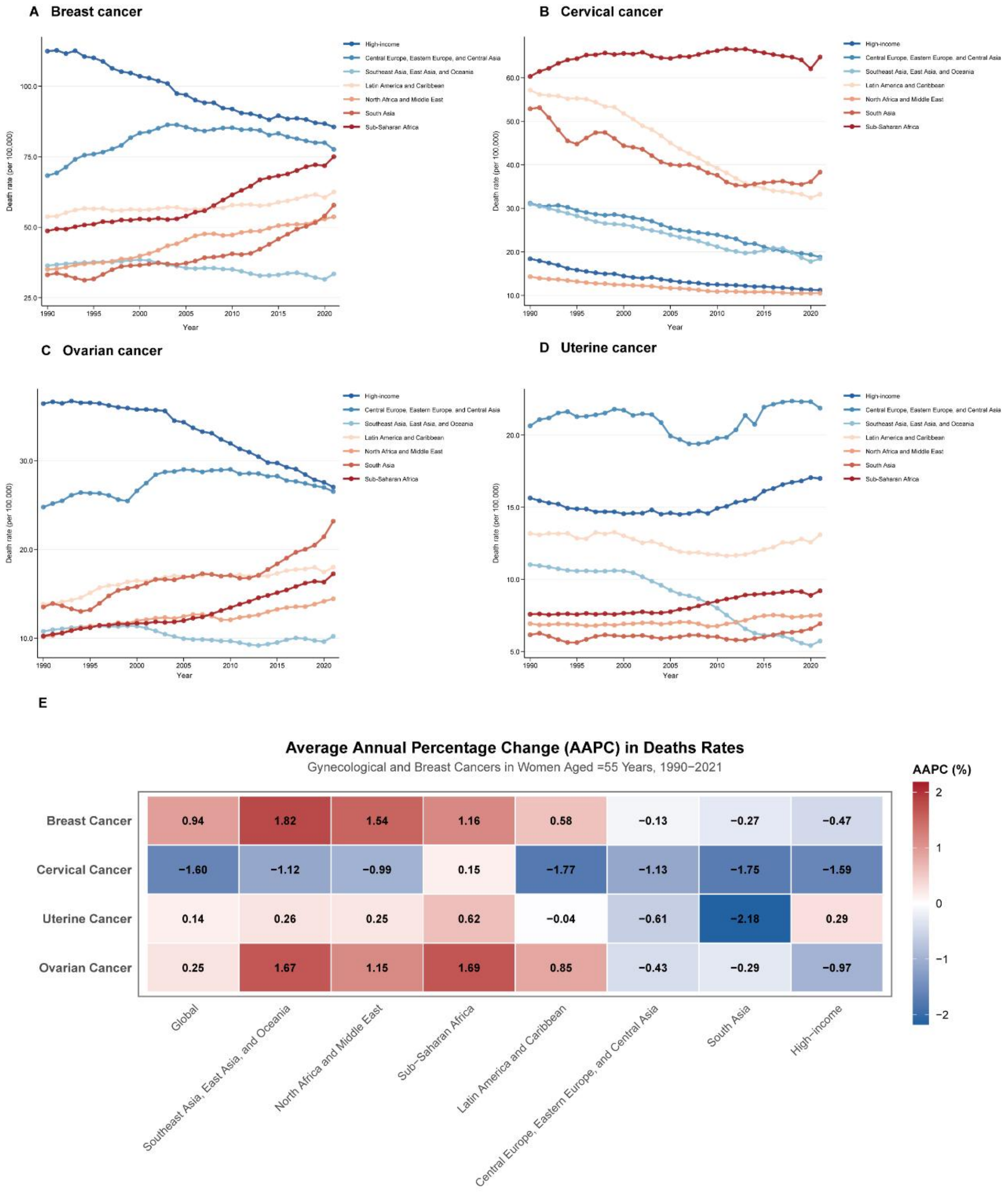

Figure S5. Association between sociodemographic index (SDI) and prevalence rate among postmenopausal women aged ≥55 years across 204 countries and territories, 1990–2021. A-D: Associations between SDI and prevalence rates for **(A)** breast cancer, **(B)** cervical cancer, **(C)** ovarian cancer, and **(D)** uterine cancer.

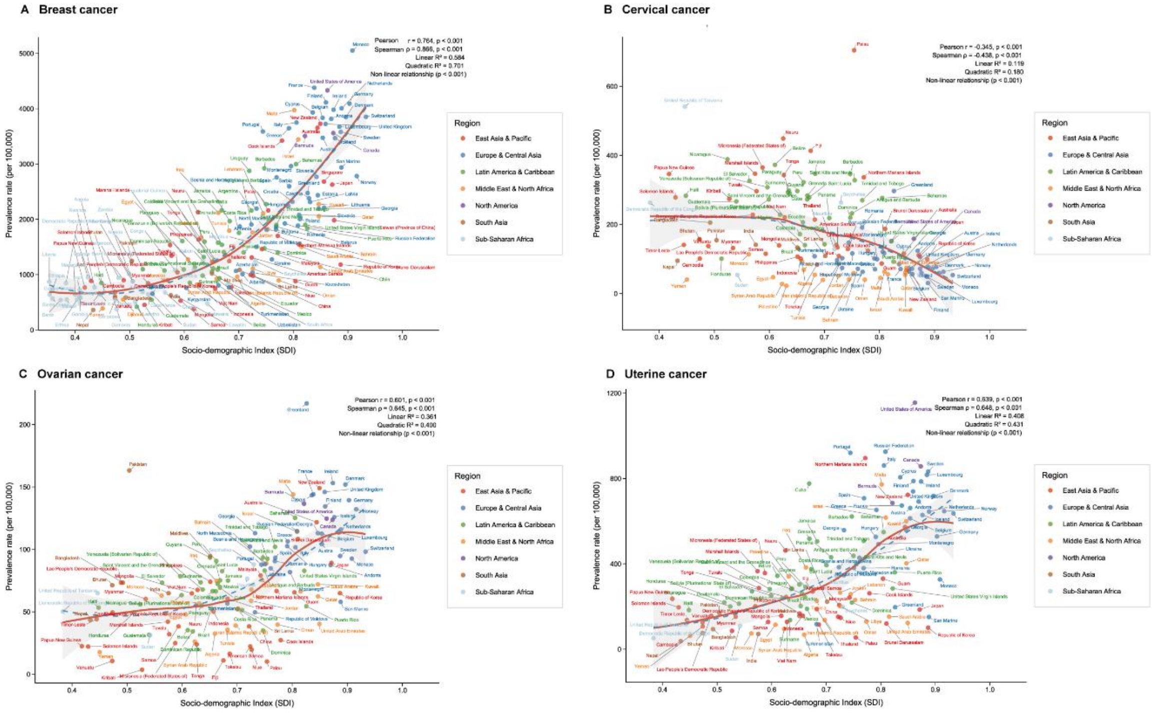

Figure S6. Association between sociodemographic index (SDI) and death rate among postmenopausal women aged ≥55 years across 204 countries and territories, 1990–2021. A-D: Associations between SDI and death rates for **(A)** breast cancer, **(B)** cervical cancer, **(C)** ovarian cancer, and **(D)** uterine cancer.

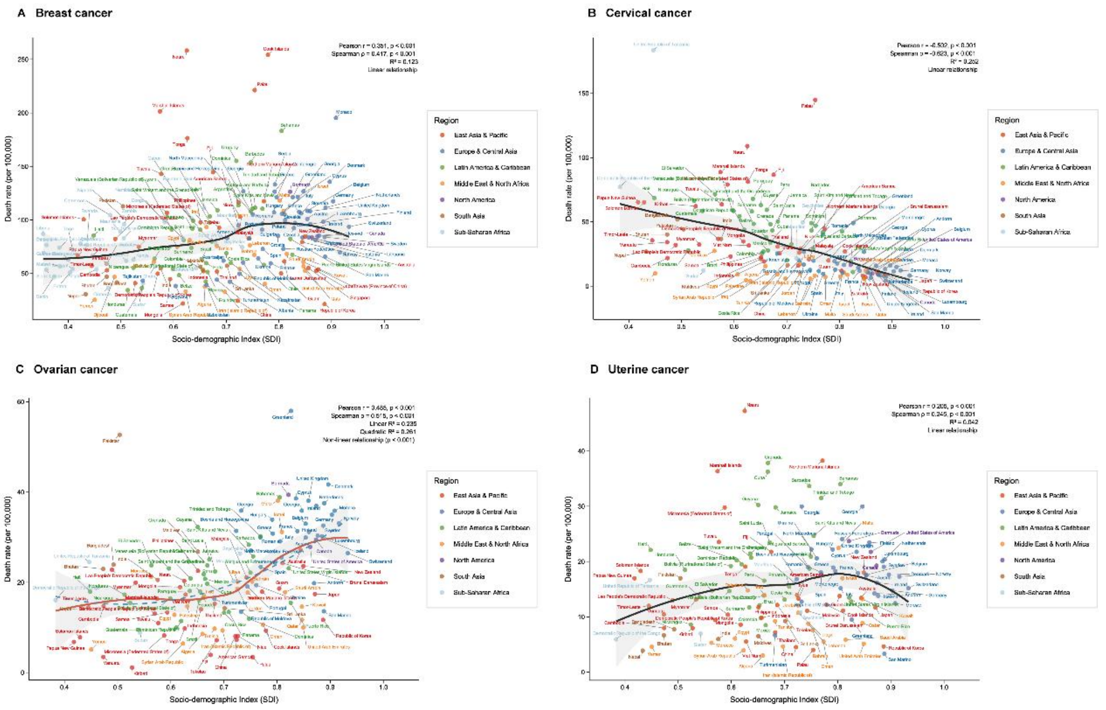

Figure S7. Health inequalities in gynecological and breast cancer prevalence rates among postmenopausal women aged ≥55 years by sociodemographic index, 1990–2021. Concentration curves showing the cumulative distribution of cancer prevalence by population ranked by sociodemographic index (SDI) for (A) breast cancer, (B) cervical cancer, (C) ovarian cancer, and (D) uterine cancer. **E-H** Slope indices of inequality showing the absolute rate difference in cancer prevalence across the SDI gradient for (E) breast cancer, (F) cervical cancer, (G) ovarian cancer, and (H) uterine cancer.

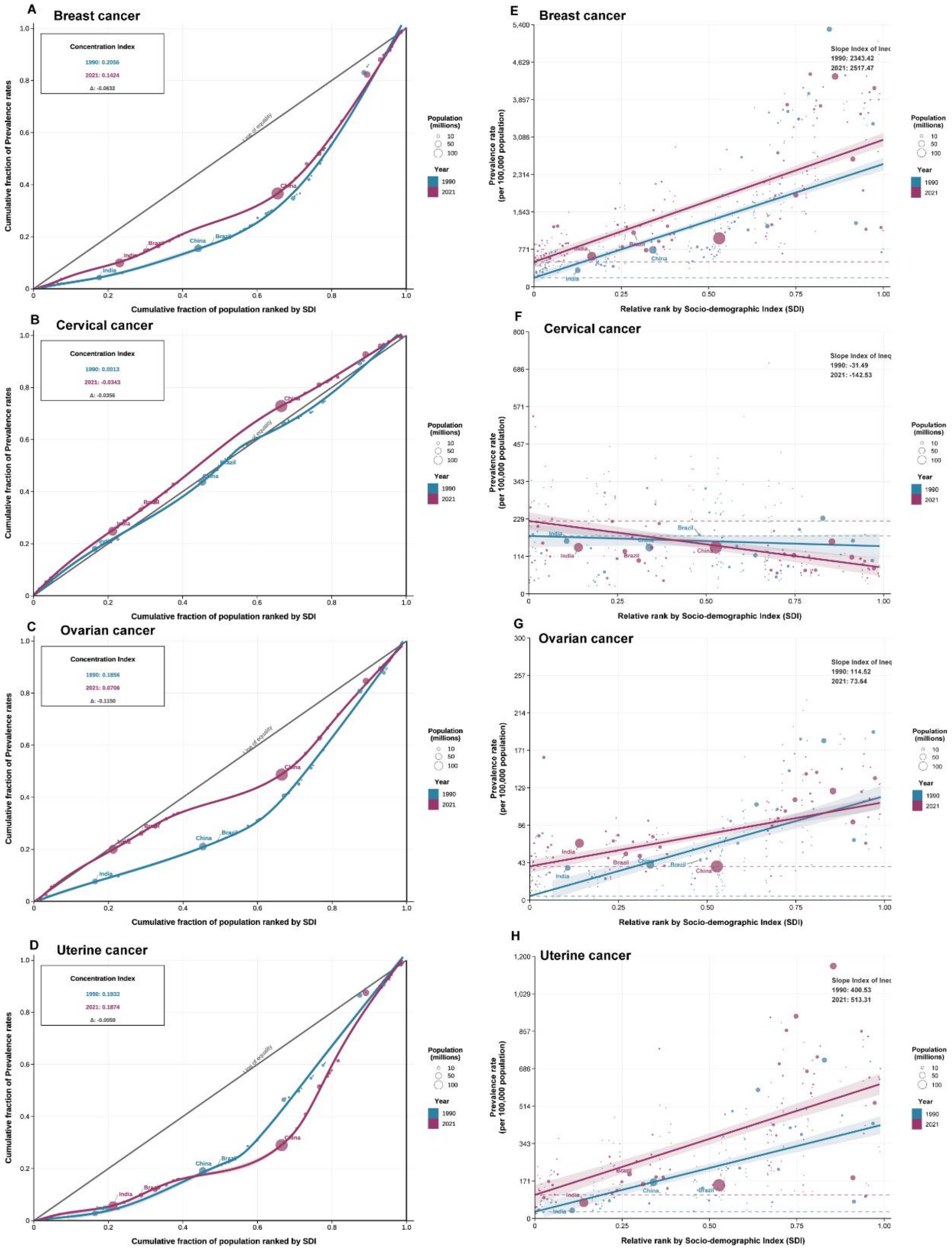

Figure S8. Health inequalities in gynecological and breast cancer DALYs rates among postmenopausal women aged ≥55 years by sociodemographic index, 1990–2021. Concentration curves showing the cumulative distribution of cancerDALYs by population ranked by sociodemographic index (SDI) for (A) breast cancer, (B) cervical cancer, (C) ovarian cancer, and (D) uterine cancer. **E-H** Slope indices of inequality showing the absolute rate difference in cancer DALYs across the SDI gradient for (E) breast cancer, (F) cervical cancer, (G) ovarian cancer, and (H) uterine cancer.

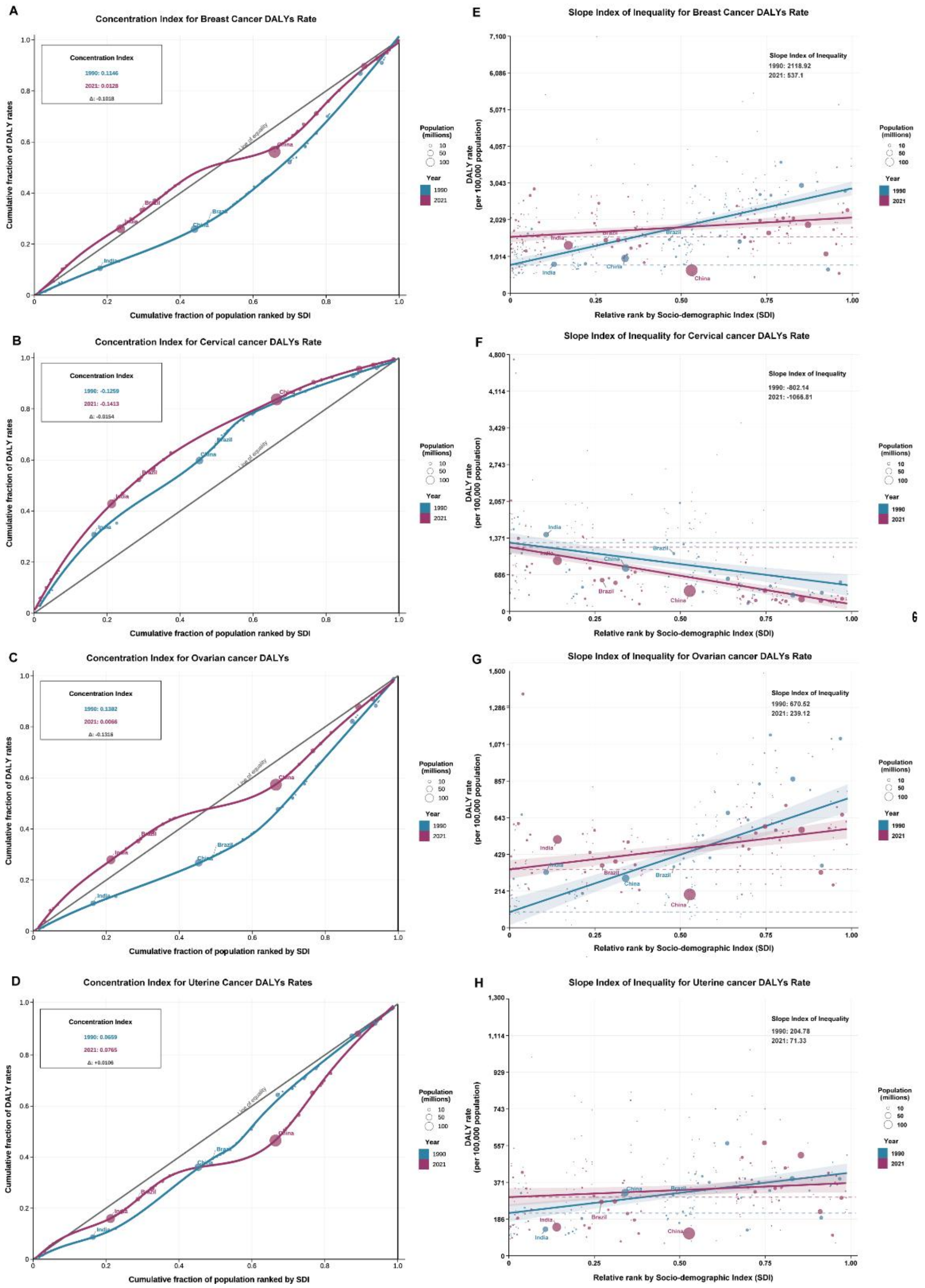

Figure S9. Health inequalities in gynecological and breast cancer death rates among postmenopausal women aged ≥55 years by sociodemographic index, 1990–2021. A-B Concentration curves showing the cumulative distribution of cancer death by population ranked by sociodemographic index (SDI) for (A) breast cancer, (B) cervical cancer, (C) ovarian cancer, and (D) uterine cancer. **E-H:** Slope indices of inequality showing the absolute rate difference in cancer death across the SDI gradient for (E) breast cancer, (F) cervical cancer, (G) ovarian cancer, and (H) uterine cancer.

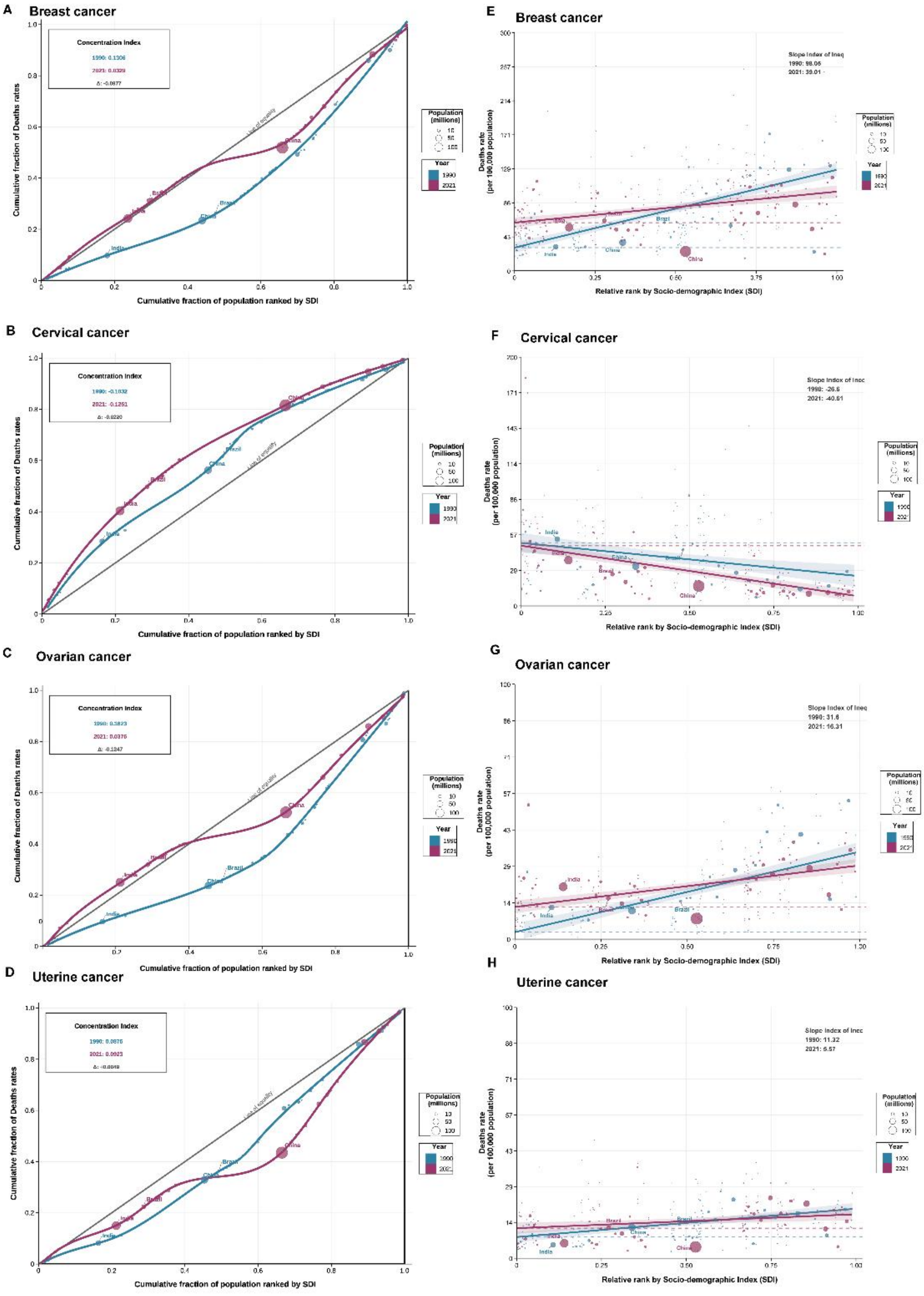

Figure S10. Projected global trends in age-standardized DALY rates of gynecological and breast cancer among postmenopausal women to 2040. Age-standardized disability-adjusted life years (DALYs) rates (per 100,000) with projections for (A) breast cancer, (B) cervical cancer, (C) ovarian cancer, and (D) uterine cancer among women aged 55 years and older globally. Shaded areas indicate uncertainty intervals (UI): the innermost darkest shading represents 80% UI and the outermost lightest shading represents 95% UI.

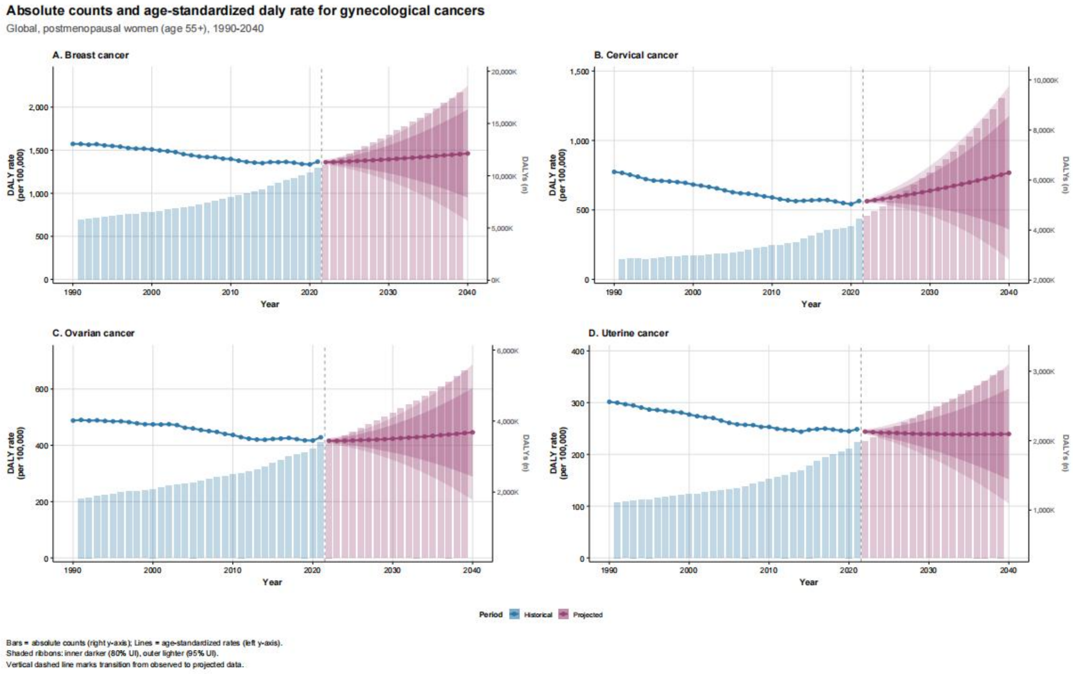

Figure S11. Sensitivity analyses for the Mendelian randomization analysis of age at menopause (ukb-b-17422) and breast cancer. A: Funnel plot for assessment of horizontal pleiotropy. B: Stability analysis of leave-one-out method. C: Scatter plot of SNP effects on exposure versus outcome. D: Leave-one-out sensitivity analysis for MR effect size using MR-Egger and IVW methods.

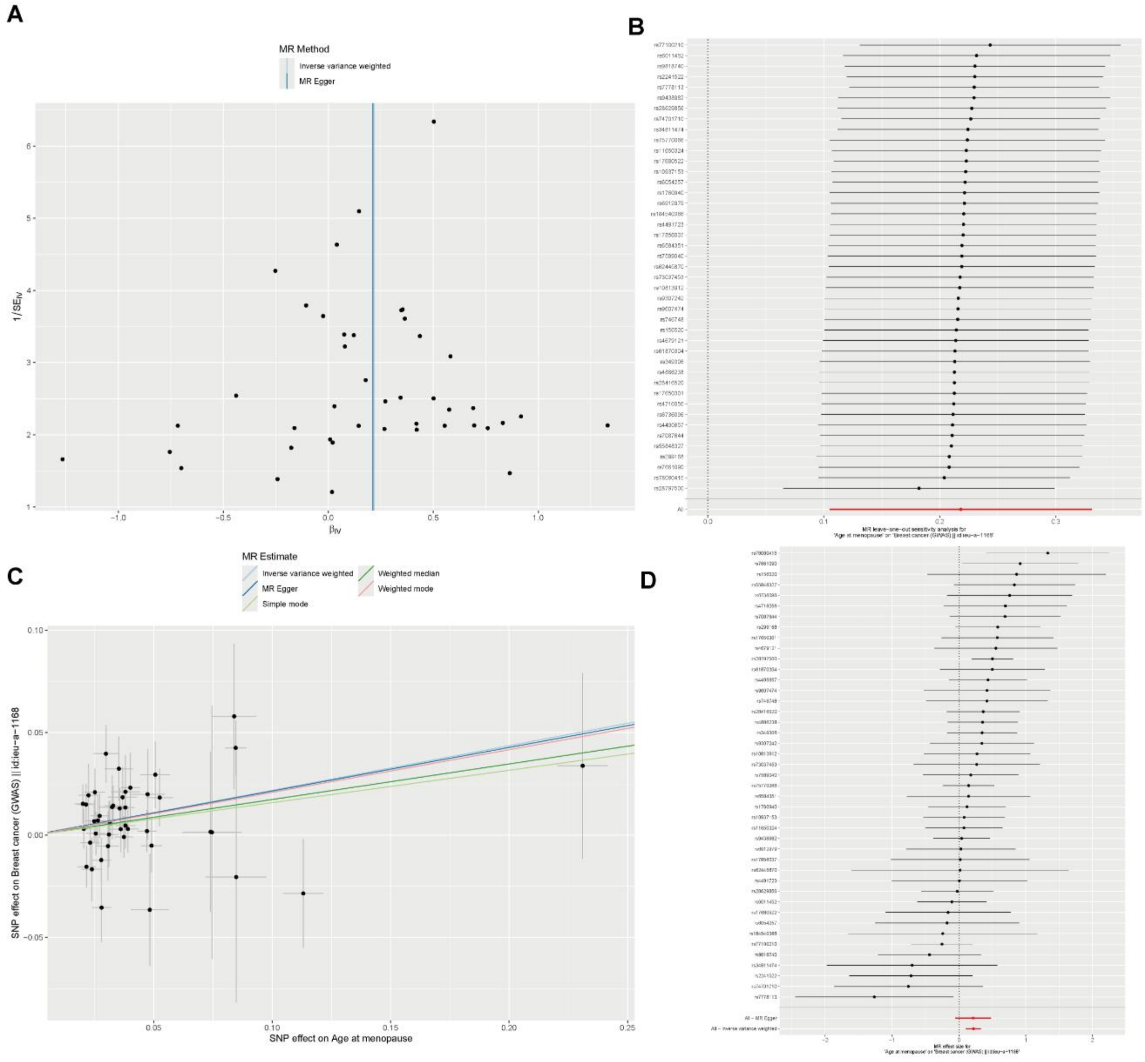

Figure S12. Sensitivity analyses for the Mendelian randomization analysis of age at menopause (ukb-b-17422) and ER- breast cancer. A: Funnel plot for assessment of horizontal pleiotropy. B: Stability analysis of leave-one-out method. C: Scatter plot of SNP effects on exposure versus outcome. D: Leave-one-out sensitivity analysis for MR effect size using MR-Egger and IVW methods.

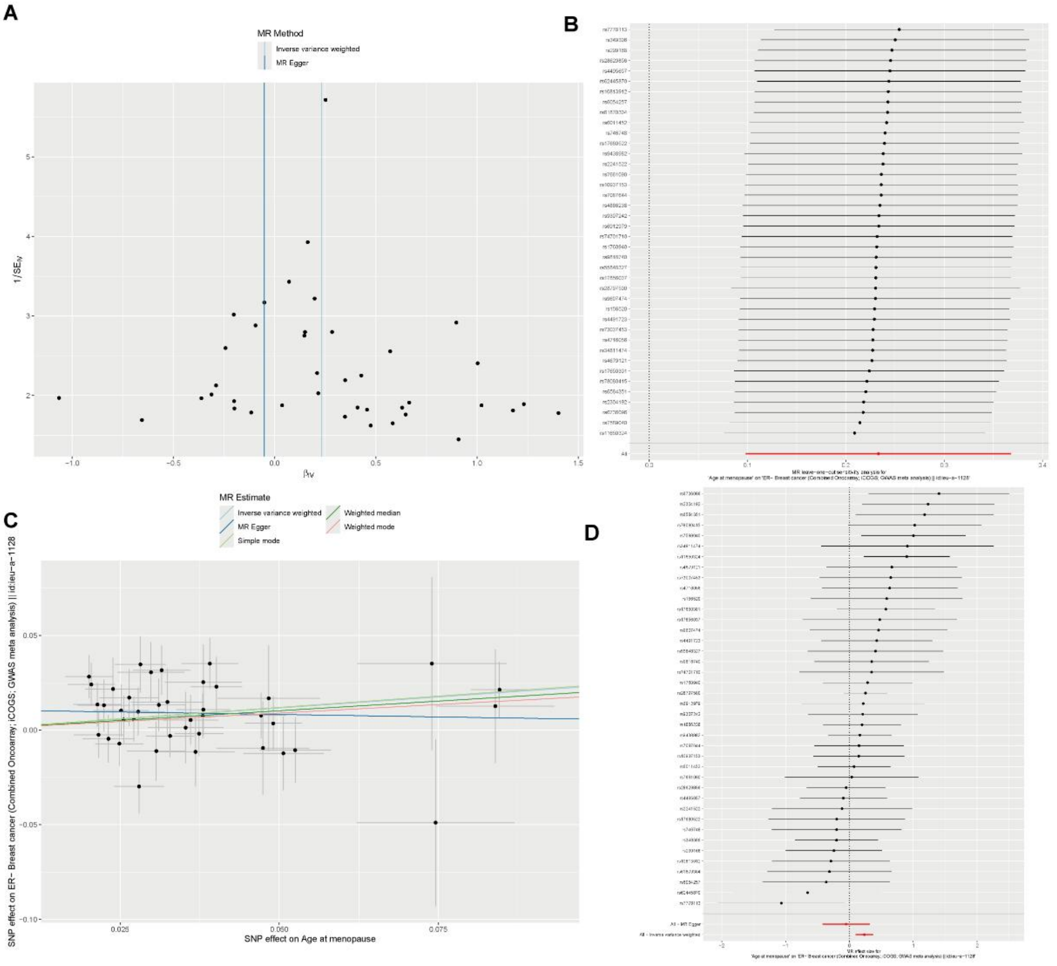

Figure S13. Sensitivity analyses for the Mendelian randomization analysis of age at menopause (ukb-b-17422) and ER+ breast cancer. A: Funnel plot for assessment of horizontal pleiotropy. B: Stability analysis of leave-one-out method. C: Scatter plot of SNP effects on exposure versus outcome. D: Leave-one-out sensitivity analysis for MR effect size using MR-Egger and IVW methods.

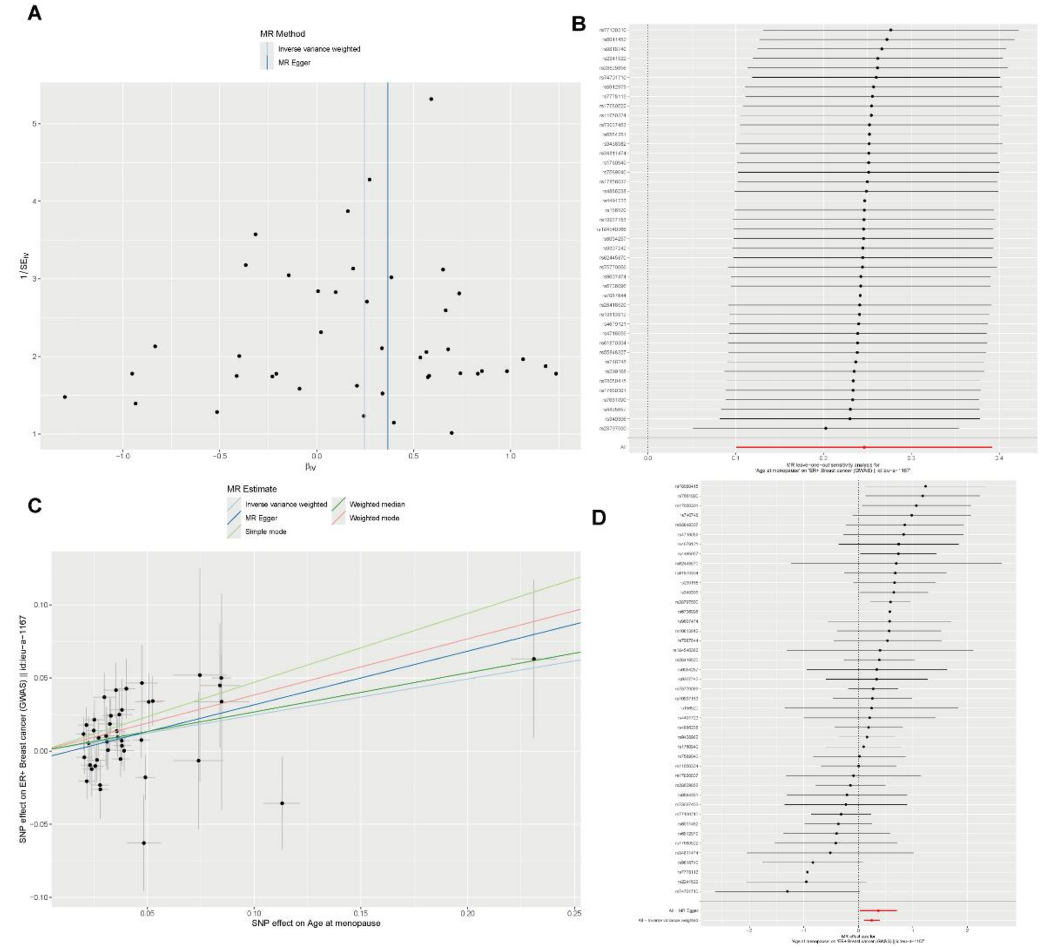

Figure S14. Sensitivity analyses for the Mendelian randomization analysis of age at menopause (ukb-b-17422) and HER2- breast cancer. A: Funnel plot for assessment of horizontal pleiotropy. B: Stability analysis of leave-one-out method. C: Scatter plot of SNP effects on exposure versus outcome. D: Leave-one-out sensitivity analysis for MR effect size using MR-Egger and IVW methods.

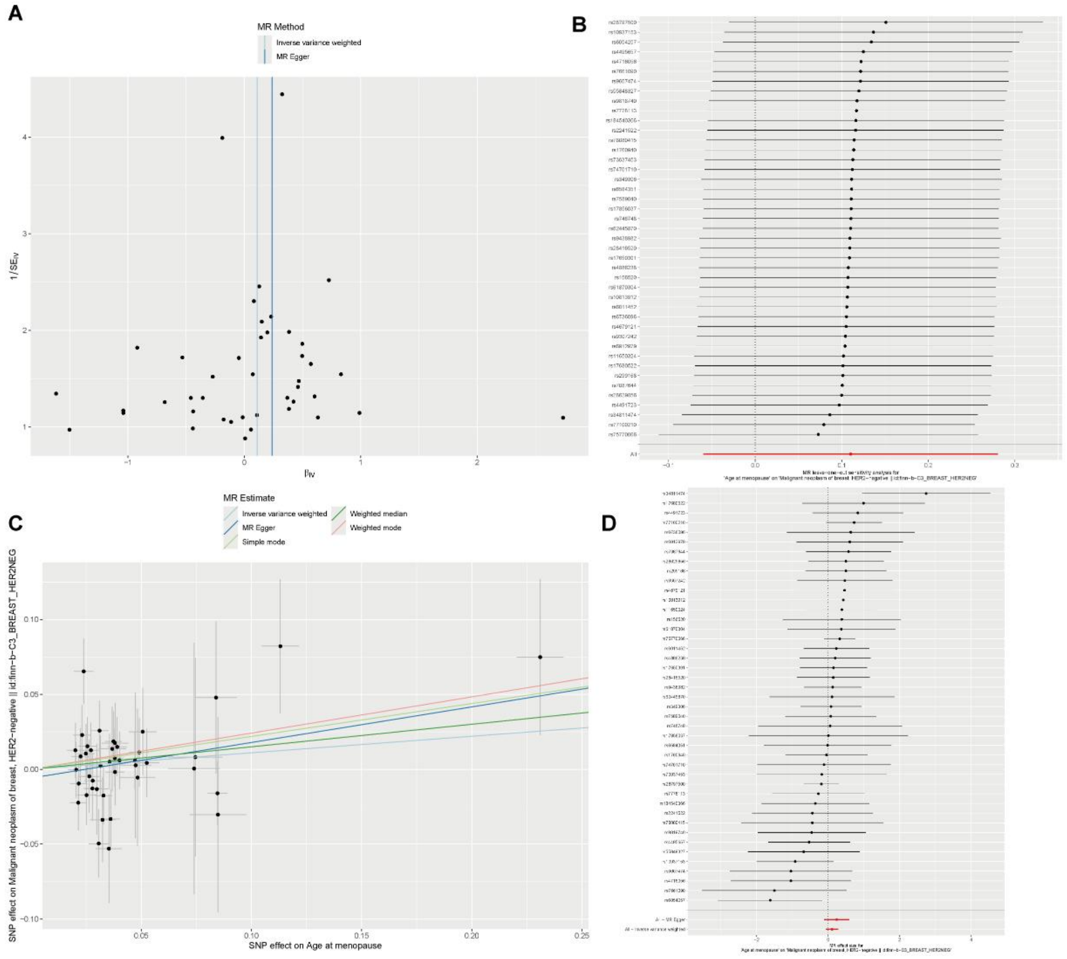

Figure S15. Sensitivity analyses for the Mendelian randomization analysis of age at menopause (ukb-b-17422) and ovarian cancer. A: Funnel plot for assessment of horizontal pleiotropy. B: Stability analysis of leave-one-out method. C: Scatter plot of SNP effects on exposure versus outcome. D: Leave-one-out sensitivity analysis for MR effect size using MR-Egger and IVW methods.

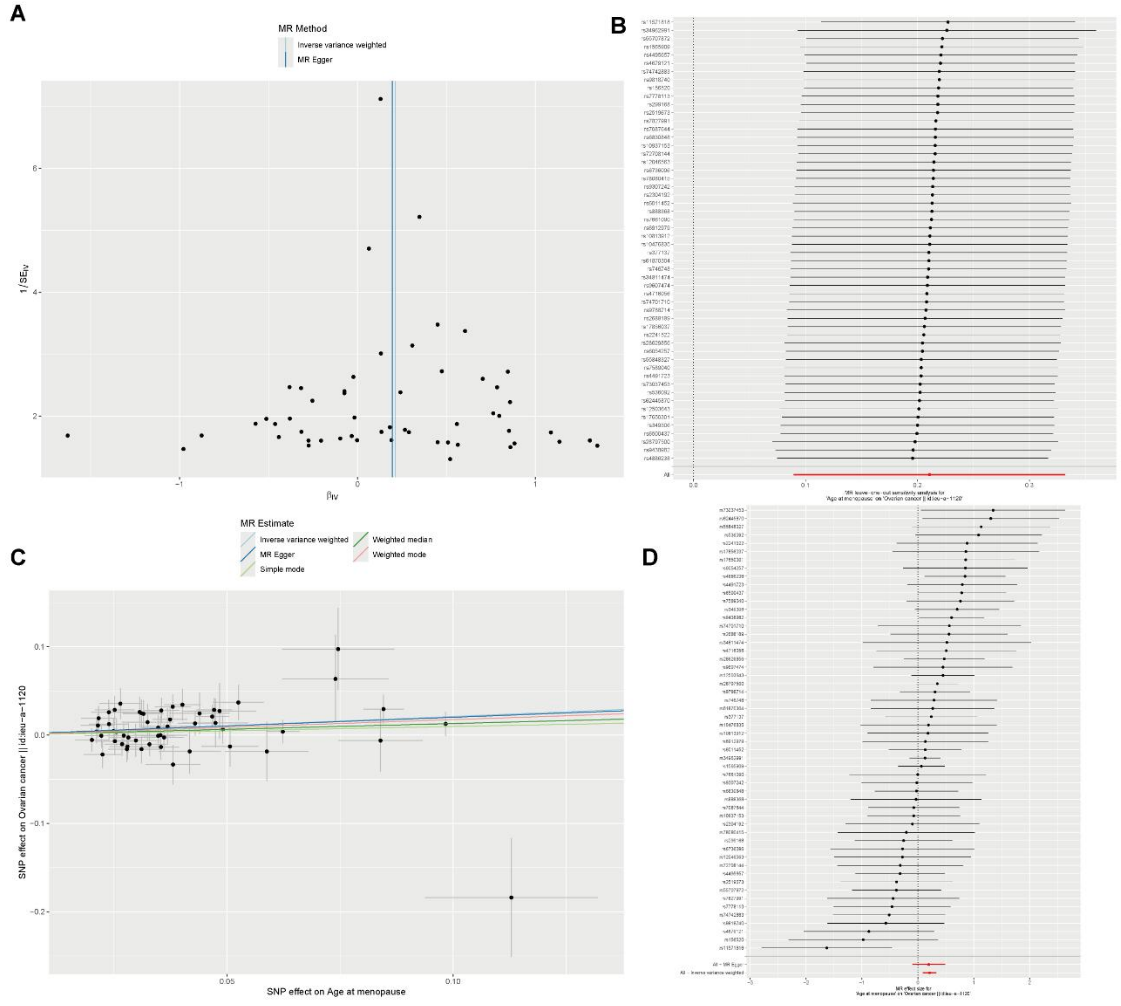

Figure S16. Sensitivity analyses for the Mendelian randomization analysis of age at menopause (ukb-b-17422) and endometrial cancer. A: Funnel plot for assessment of horizontal pleiotropy. B: Stability analysis of leave-one-out method. C: Scatter plot of SNP effects on exposure versus outcome. D: Leave-one-out sensitivity analysis for MR effect size using MR-Egger and IVW methods.

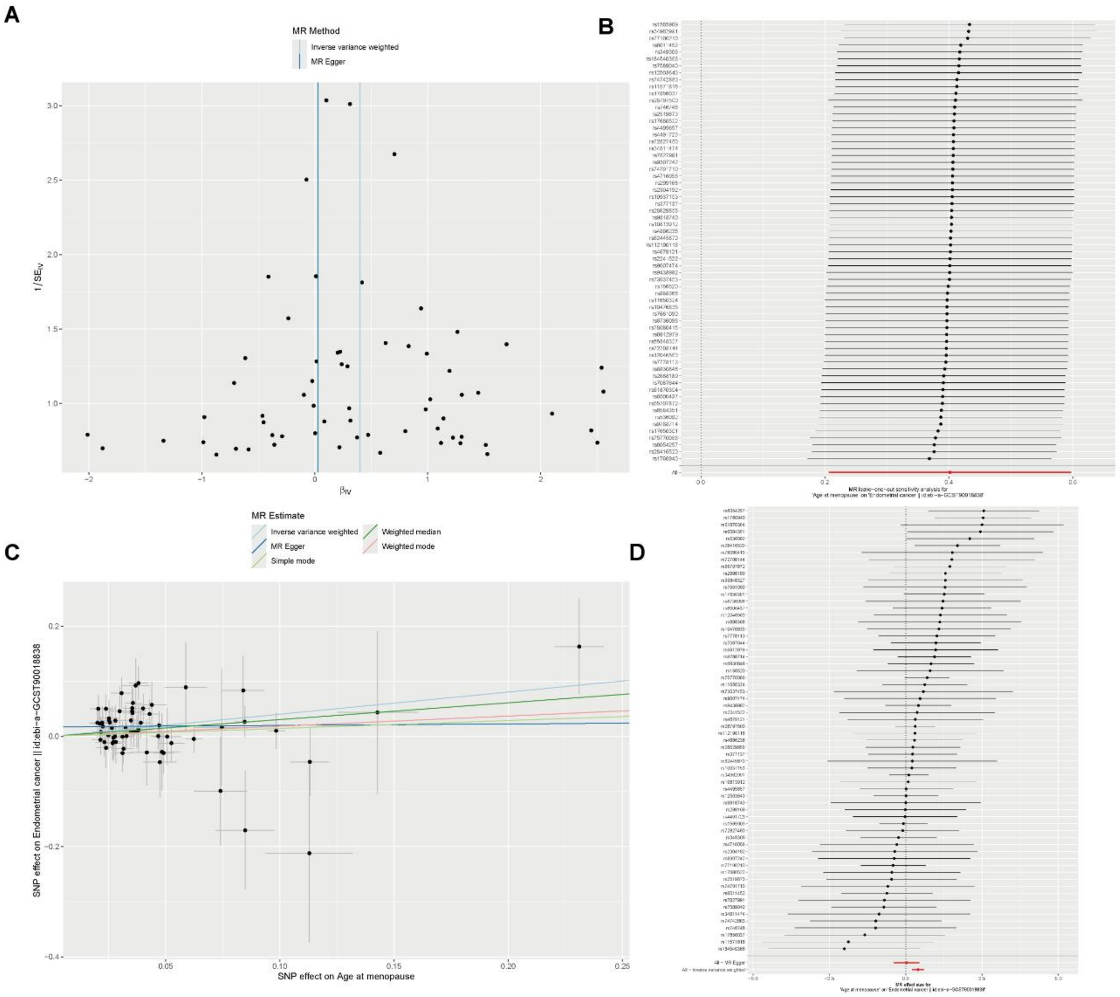

Figure S17. Sensitivity analyses for the Mendelian randomization analysis of age at menopause (ukb-b-17422) and cervical cancer. A: Funnel plot for assessment of horizontal pleiotropy. B: Stability analysis of leave-one-out method. C: Scatter plot of SNP effects on exposure versus outcome. D: Leave-one-out sensitivity analysis for MR effect size using MR-Egger and IVW methods.

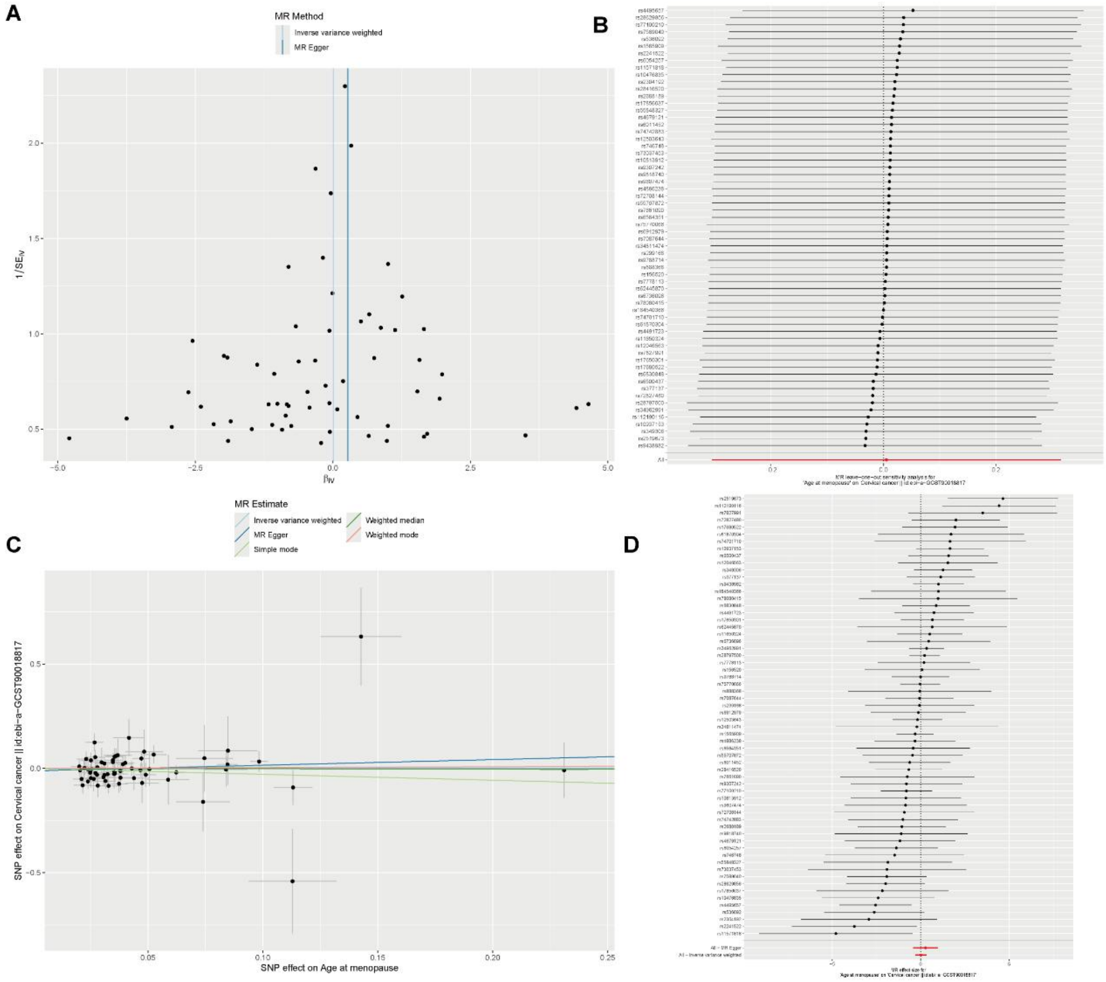

Figure S18. Sensitivity analyses for the Mendelian randomization analysis of age at menopause (ieu-a-1004) and breast cancer. A: Funnel plot for assessment of horizontal pleiotropy. B: Stability analysis of leave-one-out method. C: Scatter plot of SNP effects on exposure versus outcome. D: Leave-one-out sensitivity analysis for MR effect size using MR-Egger and IVW methods.

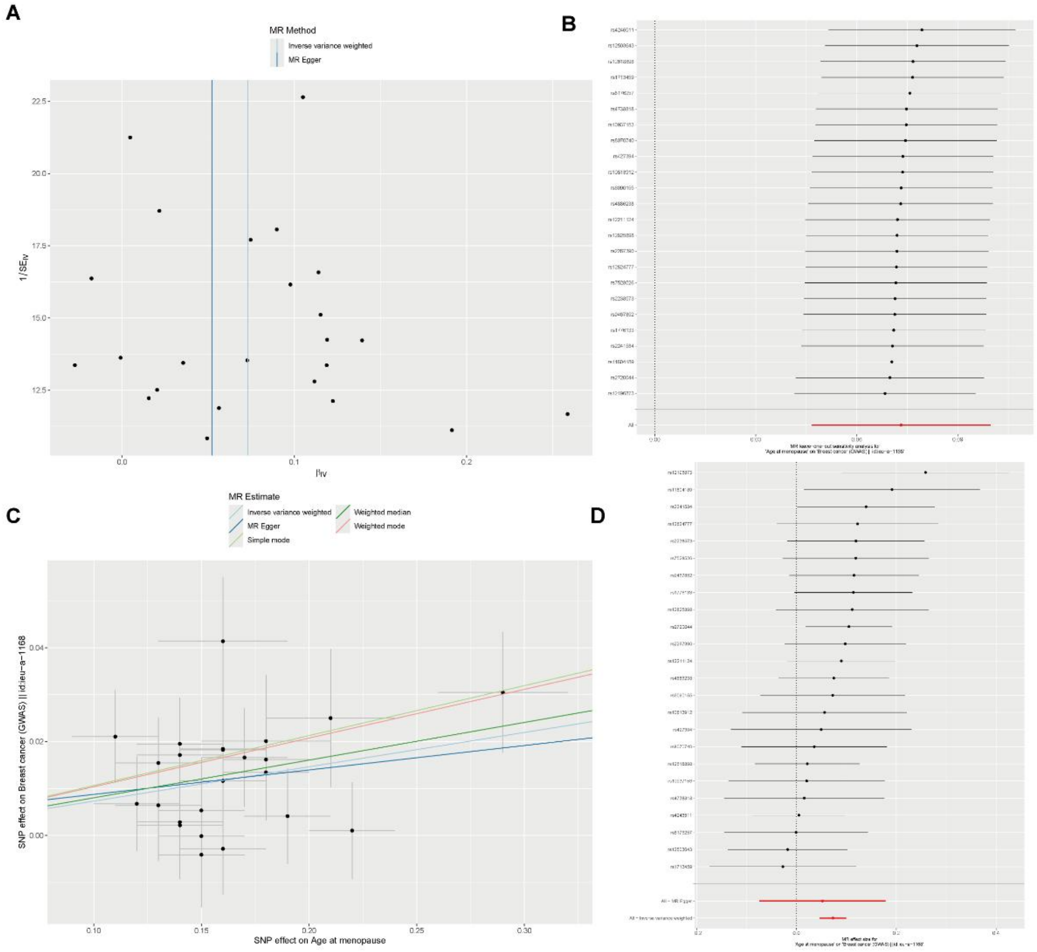

Figure S19. Sensitivity analyses for the Mendelian randomization analysis of age at menopause (ieu-a-1004) and ER- breast cancer. A: Funnel plot for assessment of horizontal pleiotropy. B: Stability analysis of leave-one-out method. C: Scatter plot of SNP effects on exposure versus outcome. D: Leave-one-out sensitivity analysis for MR effect size using MR-Egger and IVW methods.

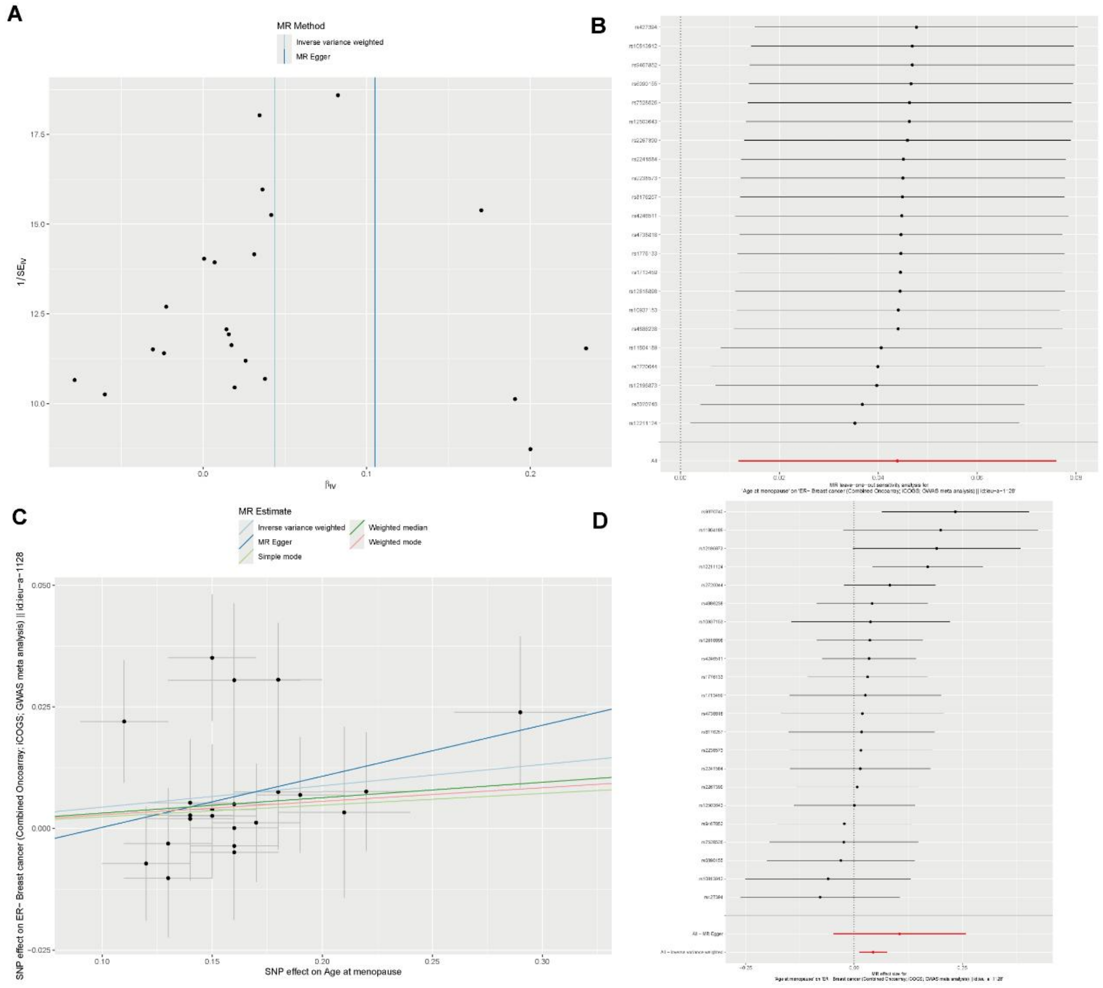

Figure S20. Sensitivity analyses for the Mendelian randomization analysis of age at menopause (ieu-a-1004) and ER+ breast cancer. A: Funnel plot for assessment of horizontal pleiotropy. B: Stability analysis of leave-one-out method. C: Scatter plot of SNP effects on exposure versus outcome. D: Leave-one-out sensitivity analysis for MR effect size using MR-Egger and IVW methods.

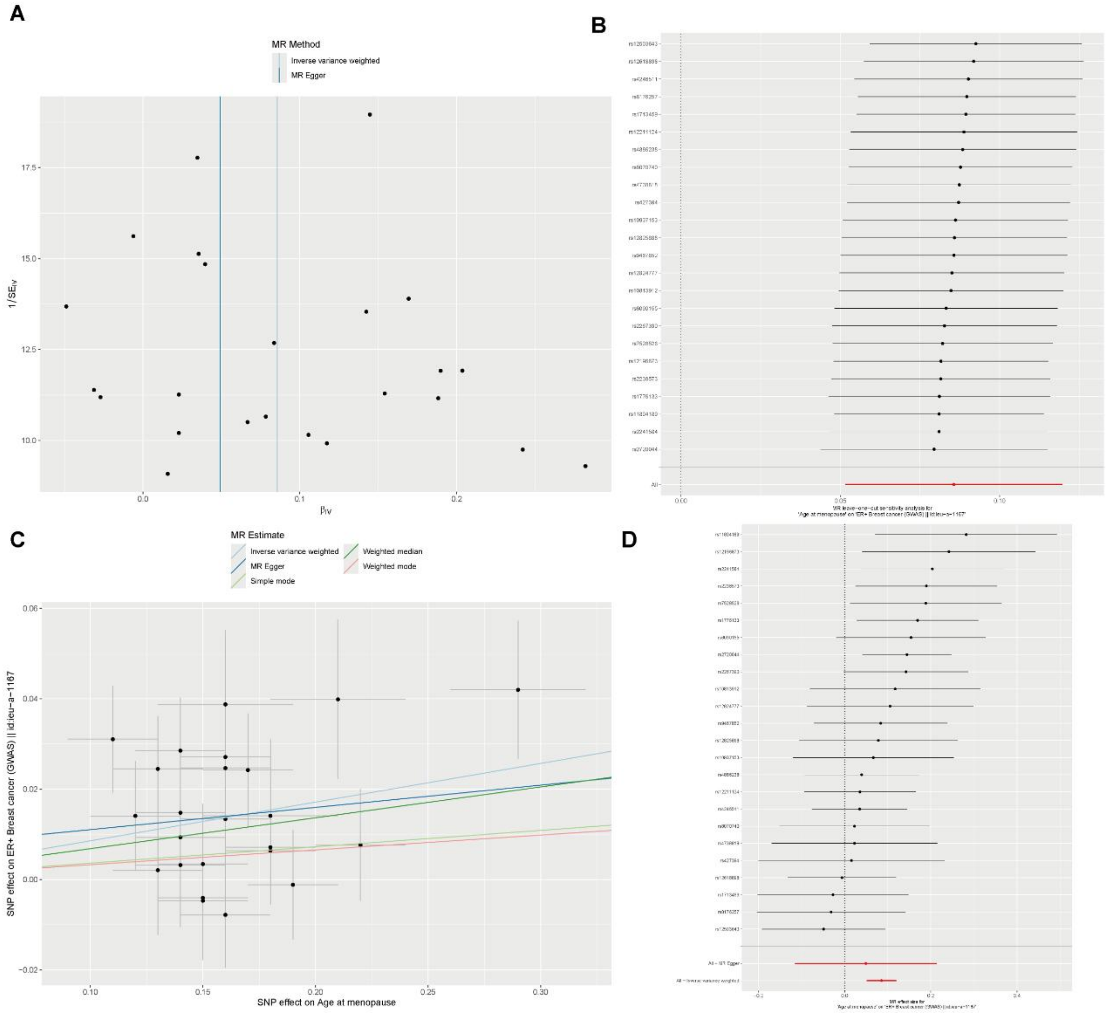

Figure S21. Sensitivity analyses for the Mendelian randomization analysis of age at menopause (ieu-a-1004) and HER2- breast cancer. A: Funnel plot for assessment of horizontal pleiotropy. B: Stability analysis of leave-one-out method. C: Scatter plot of SNP effects on exposure versus outcome. D: Leave-one-out sensitivity analysis for MR effect size using MR-Egger and IVW methods.

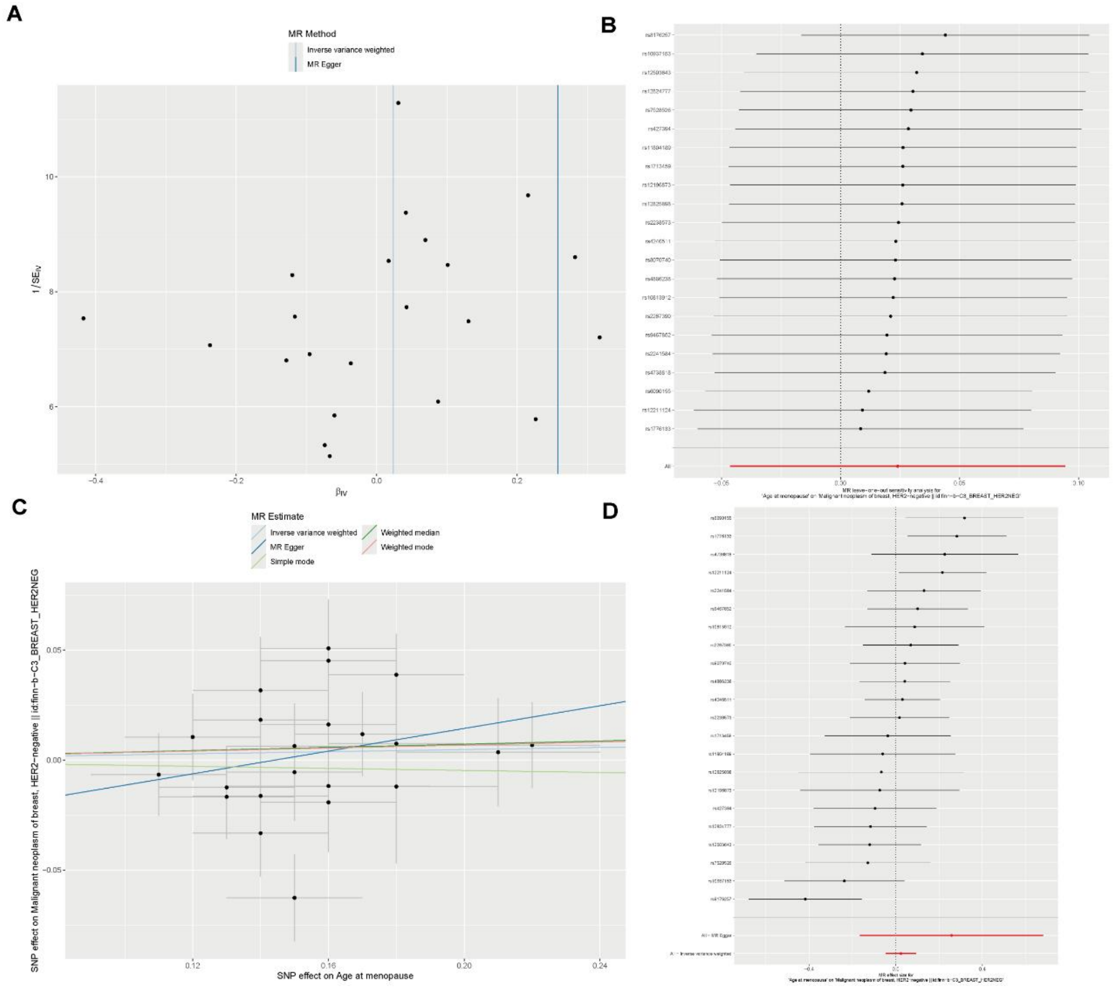

Figure S22. Sensitivity analyses for the Mendelian randomization analysis of age at menopause (ieu-a-1004) and ovarian cancer. A: Funnel plot for assessment of horizontal pleiotropy. B: Stability analysis of leave-one-out method. C: Scatter plot of SNP effects on exposure versus outcome. D: Leave-one-out sensitivity analysis for MR effect size using MR-Egger and IVW methods.

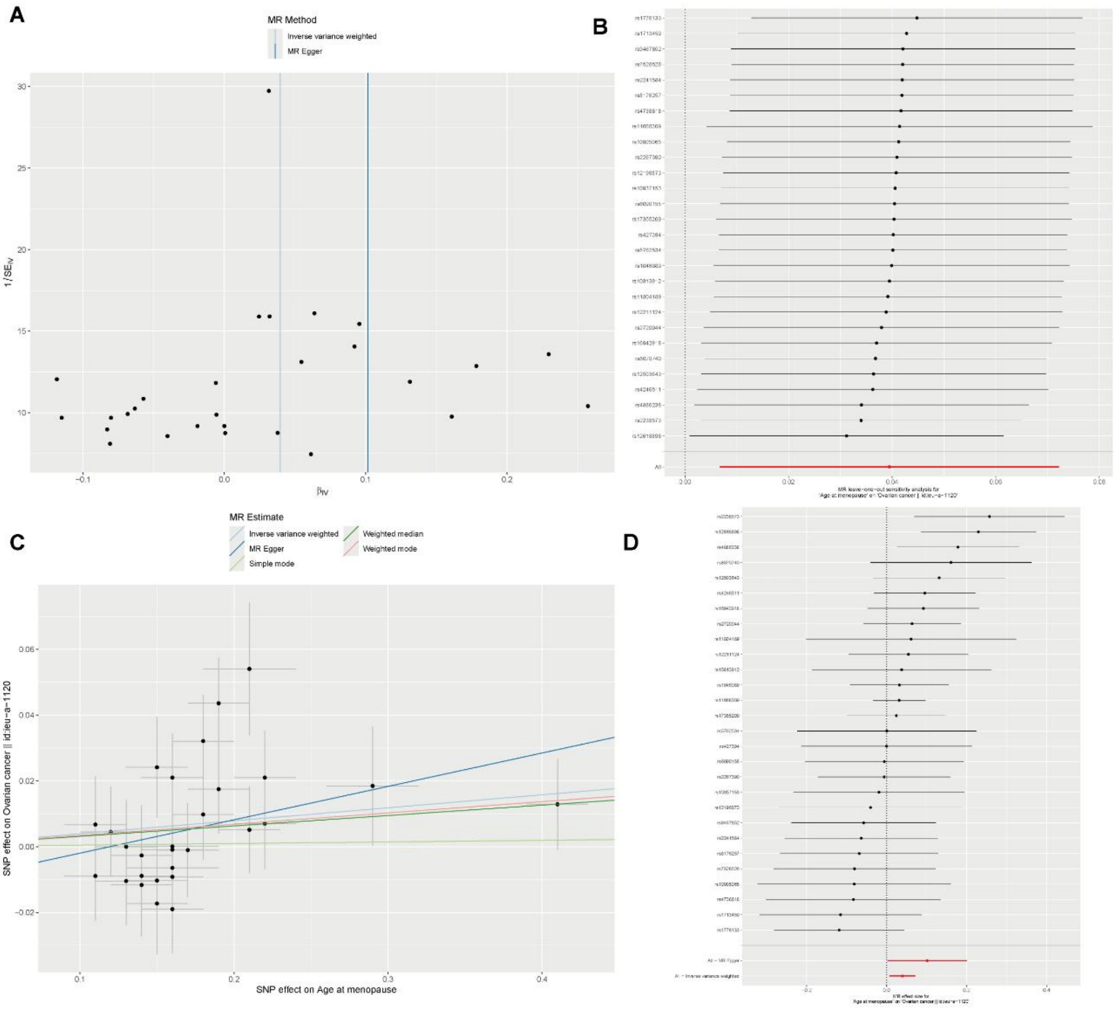

Figure S23. Sensitivity analyses for the Mendelian randomization analysis of age at menopause (ieu-a-1004) and endometrial cancer. A: Funnel plot for assessment of horizontal pleiotropy. B: Stability analysis of leave-one-out method. C: Scatter plot of SNP effects on exposure versus outcome. D: Leave-one-out sensitivity analysis for MR effect size using MR-Egger and IVW methods.

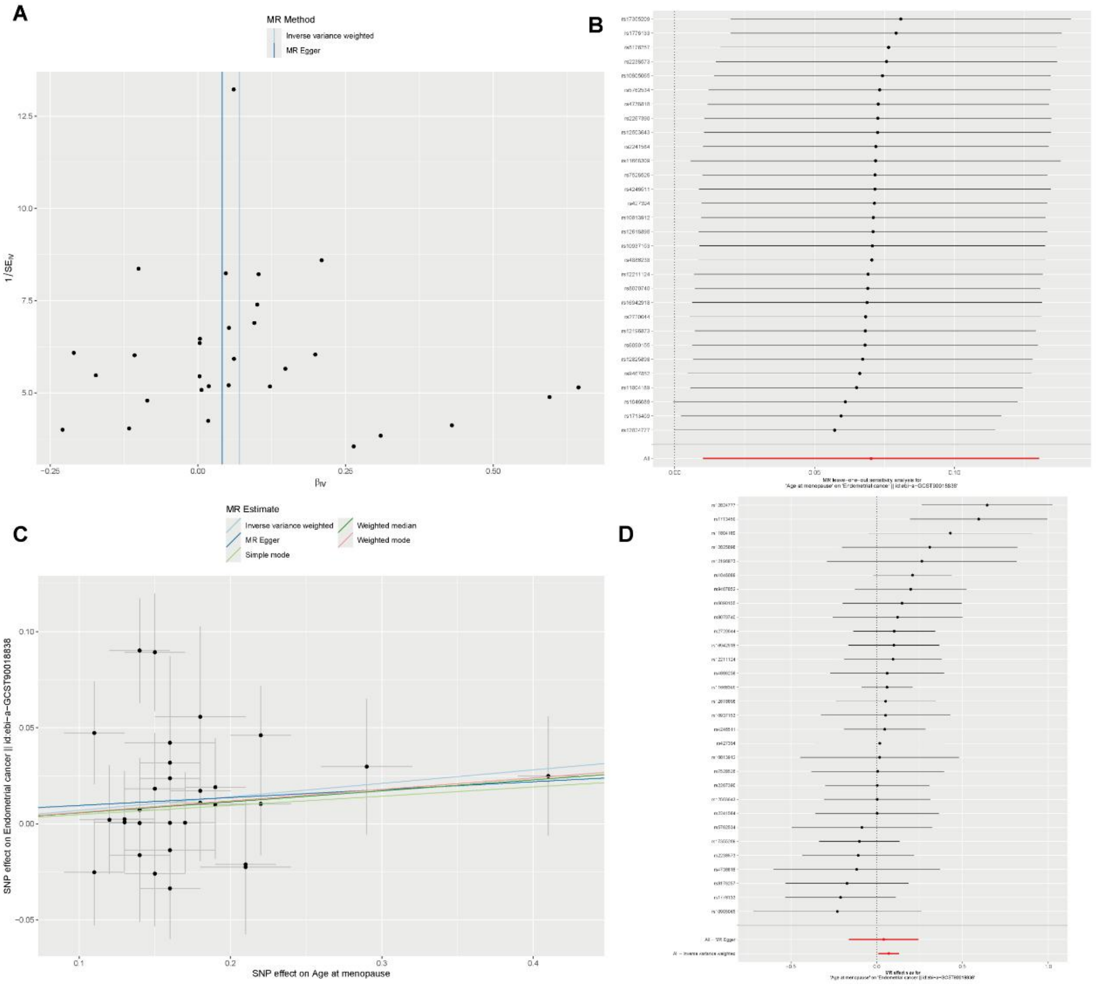

Figure S24. Sensitivity analyses for the Mendelian randomization analysis of age at menopause (ieu-a-1004) and cervical cancer. A: Funnel plot for assessment of horizontal pleiotropy. B: Stability analysis of leave-one-out method. C: Scatter plot of SNP effects on exposure versus outcome. D: Leave-one-out sensitivity analysis for MR effect size using MR-Egger and IVW methods.

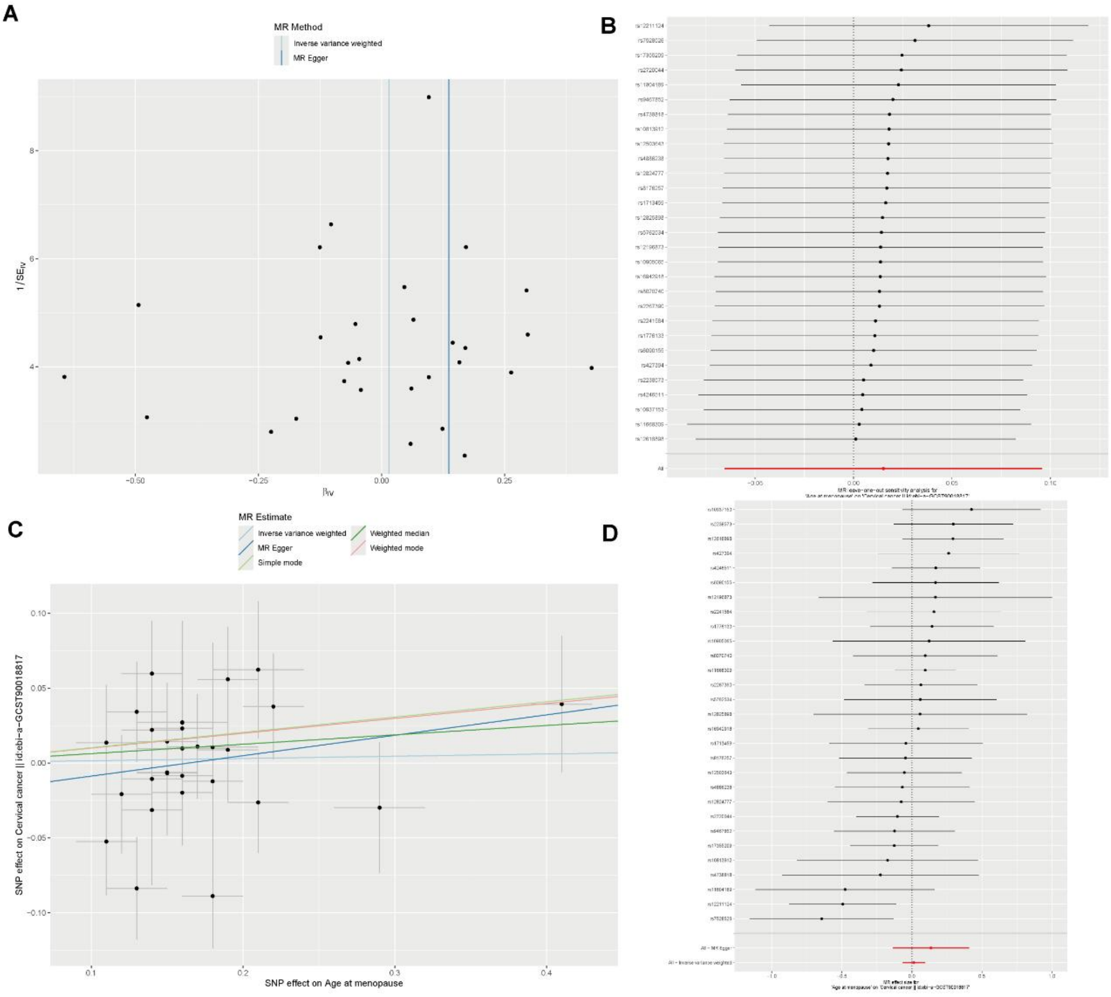

Supplement: Document S1. Figures S1–S24 [file mmc1.pdf]
